# Supplementary material for: Electrocatalytic synthesis of heterocycles from biomass-derived furfuryl alcohols
Source: Nat Commun. 2021 Mar 25;12:1868. doi: 10.1038/s41467-021-22157-5 (PMC7994825; doi:10.1038/s41467-021-22157-5)
Supplement: Supplementary file 1 — Supplementary Information [file 41467_2021_22157_MOESM1_ESM.docx]

**Supplementary Information**

Electrocatalytic synthesis of heterocycles from biomass-derived furfuryl alcohols

Xuan Liu,^a^ Bo Li,^b^ Guanqun Han,^a^ Xingwu Liu,^c^ Zhi Cao,^c,d,*^ De-en Jiang,^b,*^ and Yujie Sun^a,*^

*^a^*Department of Chemistry, University of Cincinnati, Cincinnati, Ohio 45221, United States

*^b^*Department of Chemistry, University of California, Riverside, California 92521, United States

*^c^*Syncat@Beijing, Synfuels CHINA Co., Ltd, Beijing 101407, China

*^d^*State Key Laboratory of Coal Conversion, Institute of Coal Chemistry, Chinese Academy of Sciences, Taiyuan 030001, China

**Supplementary Methods**

**Materials.** The following chemicals were purchased and used as received: NiCl_2_•6H_2_O, K_2_HPO_4_ (Fisher chemical), KH_2_PO_4_ (Amresco), acetylacetone (acac), Na_2_CO_3_ (Sigma-aldrich), cyclohexane-1,3-dione (cyacac), NaHCO_3_ (Chem-impex), AcONa (Macron fine chemical), furfuryl alcohol (Alfa Aesar), furfural (TCI), CDCl_3_ (Cambridge Isotope Laboratories). Carbon Paper (Toray 060) were purchased from Fuel Cell Store. Deionized water (18 MΩ•cm) from a Barnstead E-Pure system was used in all experiments. Ni(OTf)_2_, 5,5’-divinyl-2,2’-bipyridine, and hydropyranone used as standard sample for HPLC quantification were all synthesized in our lab (detailed procedures are shown below). All the other reagents and solvents were purchased from commercial sources and used without purification.

**Characterization.**

Scanning electron microscopy (SEM) (FEI XL30, 15 kV) was used to characterize various samples. Renishaw InVia Raman microscope was used to collect Raman spectra of various samples with laser lines at 514 nm and 633 nm. X-ray photoelectron spectroscopy (XPS) measurements were carried out on an X-ray photoelectron spectrometer (K-alpha, Thermo Fisher Scientific, USA) by using a monochromatic Al Kα X-ray source. Of the measurement, C1s peak at 284.6 eV was taken as an internal standard. The survey scans were performed using the following parameters: An energy scan range from 1,350 to -10 eV; a pass energy of 200 eV; a dwell time of 100 ms and a step size of 1 eV. For the high-resolution spectra, the scans were carried out within a pass energy of 30 eV, the number of scans was 30, and a step size of 0.1 eV with a dwell time of 50 ms. The samples are sputtered by 1 keV Ar^+^ for 60s to remove adventitious contaminants at the surface. Fourier Transform Infrared Spectrophotometer (FT-IR) Nicolet 6700 was used to collect the IR spectrum of each sample of interest in the 400 – 4,000 cm^-1^ spectral range. Renishaw InVia Raman microscope is used to collect Raman spectra at 514 nm. The ^1^H NMR spectra were collected on a Bruker Avance III HD Ascend 400 MHz NMR. The ^1^H and ^13^C NMR (400 MHz) chemical shifts were measured relative to CDCl_3_ or TMS as the internal reference (CDCl_3_: δ = 7.26 ppm; TMS: δ = 0.00 ppm).

To analyze the products of furfuryl oxidation quantitatively, 10 μL of the electrolyte solution during chronoamperometry was withdrawn from the electrolyte solution and diluted with 990 μL water, which was then analyzed using high-performance liquid chromatography (HPLC) on a Agilent 1260 infinity II system at 25 °C. The HPLC was equipped with an ultraviolet−visible detector and a 4.6 mm × 150 mm Shim-pack GWS 5 μm C_18_ column. A mixture of eluting solvents (A and B) was utilized. Solvent A was 5 mM ammonium acetate aqueous solution and solvent B was methanol. Separation and quantification were accomplished using an isocratic elution of 80% A and 20% B and the flow rate was set at 1 mL min^−1^. The identification and quantification of the products were determined from the calibration curves by applying standard solutions with known concentrations of commercially purchased pure reactants, intermediates, and final products. The conversion (%) of organic substrates and the yield (%) of oxidation products were calculated based on the following two equations:

$Conversion (\%)=\frac{mole of substrate consumed}{mole of initial substrate}\times100\%$ (eq 1)

$Yield (\%)=\frac{mole of product formed}{mole of initial substrate}\times100\%$ (eq 2)

The faradaic efficiency (FE) of product formation was calculated using the following equation:

$FE (\%) =\frac{mole of product formed}{total charge passed/(n\times F)}\times100\%$ (eq 3)

where n is the number of electron transfer for each product formation and F is the Faraday constant (96 485 C mol^−1^).

**Synthesis of monomer ligand**^[1]^

**(a) Synthesis of 5,5’-bis(bromomethyl)-2,2’-bipyridine**





5,5’-Dimethyl-2,2’-bipyridine (3.5 g, 19 mmol) was dissolved in 40 mL of tetrachloromethane. 7.1 g of N-bromosuccinimide (NBS, 40 mmol) and benzyl peroxide (BPO, 0.1 g) were added to the above solution. After being refluxed for 10 h, the solution was cooled to room temperature, and the reaction was quenched with NaHSO_3_. The solution was extracted with CHCl_3_. The organic layer was washed with NaHSO_3_ and saturated brine twice and dried over anhydrous Na_2_SO_4_. After removal of solvent, the crude product 5,5’-bis(bromomethyl)-2,2’-bipyridine was purified through silica gel with hexane and ethyl acetate =5:1 (4.7g, yield: 73%).

**(b) Synthesis of ([2,2'-bipyridine]-5,5'-diylbis(methylene))bis(triphenylphosphonium)**





5,5’-bis(bromomethyl)-2,2’-bipyridine (2.5 g, 7.3 mmol) and PPh_3_ (5.0 g, 19 mmol) were dissolved in 40 mL of DMF. The reaction mixture was stirred at 90 °C overnight and produced the light yellow bisphosphonium salt deposition. The precipitate was filtered, washed with ether, and dried under vacuum to afford bisphosphonium salt almost quantitatively.

**(c) Synthesis of 5,5’-divinyl-2,2’-bipyridine**





Bisphosphonium salt was added into a 100 mL three-necked flask. A mixture of CH_2_Cl_2_ (40mL) and 40% aqueous HCHO (12 mL) was added to the flask. The solution was cooled in ice water and stirred vigorously. 10% aqueous NaOH (20 mL) was added dropwise over 1 h with constant stirring under N_2_. The resulting mixture was stirred at room temperature overnight, and then 50 mL of water was added to the above solution. The resulting solution was extracted with CH_2_Cl_2_. The combined organic layers were washed with saturated brine twice and dried over anhydrous Na_2_SO_4._ The solvent was removed to dryness under reduced pressure to afford the mixtures. The mixtures were purified on silica column using the mixed solvents of haxane and ethyl acetate = 20:1 as an eluent to afford a white solid (1.3 g, 98% yield) and was kept in the dark at - 4 °C before using.

**Synthesis of nickel(II) trifluoromethanesulfonate.** ^[2]^

Trifluoromethanesulfonic acid (2.6 mL, 30 mmol) was added dropwise to a suspension of NiCO_3_ (1.42 g, 12 mmol) in CH_3_CN (60 mL) under vigorous stirring. The above mixture was stirred at 50 °C for 5 h under N_2_. Then the solvent was removed under vacuum. The mud was washed with diethyl ether. Solid was obtained after recrystallization with CH_3_CN and diethyl ether and dried at 70 °C under vacuum to afford Ni(OTf)_2_ as a light green solid (3.75 g, yield: 87%).

**Synthesis of 6-Hydroxy-2H-pyran-3(6H)-one (2a)**

Synthesis 1: To a solution of furfuryl alcohol (0.3 g, 3 mmol), NaHCO_3_ (0.52 g, 6 mmmol) and NaOAc (0.3 g, 3 mmol) in THF/H_2_O (4:1, 10 ml) was added NBS (0.6 g, 3.2 mmol) in ice bath. After 2 h, the reaction mixture was extracted with ethyl acetate. The combined organic phase was dried over Na_2_SO_4_ and then removed the solvent. The crude product was purified through silica gel with ethyl acetate: hexane=3:1-1:1 to afford pale yellow oil (0.25 g, yield: 73%).^[3]^

Synthesis 2: To a solution of furfuryl alcohol (0.9 g, 9 mmol) in 10 mL dichloromethane at 0 °C was added *m*-CPBA (1.2 g, 1.35 mmol). The reaction mixture was stirred at ambient temperature for 5 h. The reaction was then cooled to -20 °C and stirred for 1 h before removal of insoluble m-chlorobenzoic acid (white precipitate) by filtration. The filtrate was concentrated in vacuo and purified by flash column chromatography with ethyl acetate: hexane=3:1-1:1 to afford colorless oil (0.85 g, 82%).^[4]^

**Syntheses of furfuryl alcohol derivatives (1c-1i)**

To a stirred solution of furan (1.86 g, 30 mmol) in diethyl ether (50 mL) at -78 °C, n-BuLi (25.0 mL 1.0 M solution in hexanes, 25 mmol) was added dropwise and then the mixture was cooled to -20 °C. After stirring for another 30 minutes, the corresponding ketone (20 mmol) was added dropwise and the resulting solution was stirred overnight. Subsequently, water (5 mL) was added to quench the reaction and THF was removed under vacuum. The mixture was extracted with diethyl ether and the organic layers were dried with Na_2_SO_4_. The crude product was purified through column chromatography with hexane and ethyl acetate.^[5]^

**2-(Furan-2-yl)propan-2-ol (1c)** The pure product was collected as colorless oil (2.14 g, 82%). ^1^H NMR (400 MHz, CDCl_3_) δ 7.37 (s, 1H), 6.32 (dt, *J* = 3.1, 1.5 Hz, 1H), 6.21 (d, *J* = 3.2 Hz, 1H), 1.61 (d, *J* = 1.3 Hz, 6H).

**1-(Furan-2-yl)butan-1-ol (1d)** The pure product was collected as colorless oil. (3.1 g, 88%). ^1^H NMR (400 MHz, CDCl_3_) δ 7.39 (s, 1H), 6.40 – 6.30 (m, 1H), 6.25 (d, *J* = 3.1 Hz, 1H), 4.71 (t, *J* = 6.9 Hz, 1H), 1.85 (t, *J* = 7.5 Hz, 2H), 1.43 (dt, *J* = 38.3, 7.0 Hz, 2H), 0.97 (t, *J* = 7.4 Hz, 3H).

**1-(Furan-2-yl)-2-phenylethan-1-ol (1e)** The pure product was collected as colorless oil (3.19 g, 75%).  ^1^H NMR (400 MHz, CDCl_3_) δ 7.42 – 7.05 (m, 6H), 6.26 (dd, *J* = 3.4, 1.8 Hz, 1H), 6.15 (d, *J* = 3.1 Hz, 1H), 4.85 (dd, *J* = 8.0, 5.6 Hz, 1H), 3.19 – 2.97 (m, 2H).

**1-(Furan-2-yl)-3-phenylpropan-1-ol (1f)** The pure product was collected as colorless oil (2.82 g, 75%). ^1^H NMR (400 MHz, ) δ 7.45 – 7.12 (m, 6H), 6.36 (q, *J* = 2.3 Hz, 1H), 6.31 – 6.20 (m, 1H), 4.71 (t, *J* = 7.1 Hz, 1H), 2.88 – 2.59 (m, 2H), 2.32 – 2.11 (m, 2H).

**Furan-2-yl(phenyl)methanol (1g)** The pure product was collected as colorless oil (2.34 g, 67%). ^1^H NMR (400 MHz, CDCl_3_) δ 7.54 – 7.32 (m, 6H), 6.34 (dt, *J* = 3.3, 1.9 Hz, 1H), 6.14 (d, *J* = 2.8 Hz, 1H), 5.86 (s, 1H).

**Furan-2-yl(p-tolyl)methanol (1h)** The pure product was collected as colorless oil (3.29 g, 87%). ^1^H NMR (400 MHz, CDCl_3_) δ 7.41 (s, 1H), 7.37 – 7.31 (m, 2H), 7.21 (d, *J* = 7.7 Hz, 2H), 6.34 (p, *J* = 1.8 Hz, 1H), 6.15 (d, *J* = 3.2 Hz, 1H), 5.85 – 5.77 (m, 1H), 2.37 (d, *J* = 11.1 Hz, 3H).

**(4-Fluorophenyl)(furan-2-yl)methanol (1i)** The pure product was collected as colorless oil (2.88 g, 75%). ^1^H NMR (400 MHz, CDCl_3_) δ 7.52 – 7.38 (m, 2H), 7.08 (t, *J* = 8.6 Hz, 2H), 6.35 (t, *J* = 2.5 Hz, 1H), 6.13 (d, *J* = 3.2 Hz, 1H), 5.85 (s, 1H), 2.42 (d, *J* = 4.0 Hz, 1H).

**(5-(4-chlorophenyl)furan-2-yl)methanol (1k)** To 5-(4-chlorophenyl)furan-2-carbaldehyde (0.21 g, 1 mmol) in 10 mL MeOH at 0 °C, NaBH_4_ (60 mg, 1.5 mmol) was added. The solution was stirred at room temperature for 1 h and then quenched with water. The solvent was removed under vacuum and the residue was extracted with CH_2_Cl_2_. The organic layer was concentrated at reduced pressure and purified through column chromatography with hexane and ethyl acetate (190 mg, 92%). (^1^H NMR (400 MHz, CDCl_3_) δ 7.62 (d, *J* = 8.1 Hz, 2H), 7.37 (d, *J* = 8.1 Hz, 2H), 6.61 (d, *J* = 3.3 Hz, 1H), 6.41 (d, *J* = 3.4 Hz, 1H), 4.69 (s, 2H).^[6]^

**5-Hydroxy-5-(2-furyl)-1-pentene (1m)** To a suspension of magnesium turnings (335 mg, 13.8 mmol) in diethyl ether (20 mL) was added 1-bromo-3-pentene (1.70 g, 12.5 mmol) under N_2_ over 30 min. The mixture was stirred at room temperature for 1 h. The resulting solution was cooled to 0 ˚C before 2-furylcarbaldehyde (1.0 g, 10.4 mmol) was introduced at that temperature. After 2 h, the mixture was quenched with HCl (1 M) and the aqueous layer was repeatedly extracted with Et_2_O. Subsequently, the combined ethereal phases were washed with brine, dried over Na_2_SO_4_, filtered, and evaporated. The pure product was purified by column chromatography (1.17 g, 78%). ^1^H NMR (400 MHz, CDCl_3_) δ 7.40 (t, *J* = 2.8 Hz, 1H), 6.40 – 6.20 (m, 2H), 5.94 – 5.73 (m, 1H), 5.12 – 4.95 (m, 2H), 4.71 (q, *J* = 6.1 Hz, 1H), 2.14 (d, *J* = 7.2 Hz, 2H), 1.97 – 1.80 (m, 3H).^[7]^

**General procedures for electrochemical Achmatowicz reaction:**

***Method A***: A 20 mL two-compartment cell was equipped with a magnetic stir bar in the anode chamber. Carbon paper loaded with Ni-DVBP and Ag/AgCl were used as the anode and reference electrodes, respectively. A carbon rod was placed in the counter chamber as the counter electrode. To the anode chamber was added 10 mL 0.1 M phosphate buffer (pH 7) and 8.7 µL furfuryl alcohol. The reaction mixture was then purged with nitrogen gas for 5 minutes. Electrolysis was performed at 1.4 V vs Ag/AgCl. The reaction was monitored by HPLC and the product yield was calculated based on obtained HPLC spectra.

***Method B***: A 20 mL two-compartment cell was equipped with a magnetic stir bar in the anode chamber. Carbon paper loaded with Ni-DVBP and Ag/AgCl were used as the anode and reference electrodes, respectively. A carbon rod was placed in the counter chamber as the counter electrode. To the anode chamber was added 10 mL 0.1 M phosphate buffer (pH 7) and 0.5 mmol furfuryl alcohol (or its derivatives). For those substrates with low solubility in phosphate buffer, 30% (v/v) THF was added to the electrolyte. The reaction mixture was then purged with nitrogen gas for 5 minutes. Electrolysis was performed at 1.4 V vs Ag/AgCl. Upon full consumption of starting material as determined by thin-layer chromatography (TLC, stained with KMnO_4_ solution), the reaction mixture was extracted with CH_2_Cl_2_ thoroughly. The product solution was concentrated under vacuum and purified with preparative TLC plate (eluted with hexanes/ethyl acetate) to yield the final product.

***Method C***: A flow electrolyzer was used for large scale synthesis. Three carbon paper electrodes loaded with Ni-DVBP were used as the anode and a Ni foam was used as the counter electrode which were separated by an anion exchange membrane. A solution (250 mL) of 0.1 M phosphate buffer and 50 mM furfuryl alcohol was pumped into the anode chamber and blank phosphate buffer into the counter chamber at a flow rate of 3 mL/h. This was a two-electrode configuration electrolysis and the cell voltage was set at 3.5 V. The reaction mixture from the outlet was collected and extracted with CH_2_Cl_2_ thoroughly. The product solution was concentrated under vacuum and purified with column chromatography (eluted with hexanes/ethyl acetate) to obtain the final product with a gram-scale yield (1.14 g, yield = 81%).

**Supplementary figures**


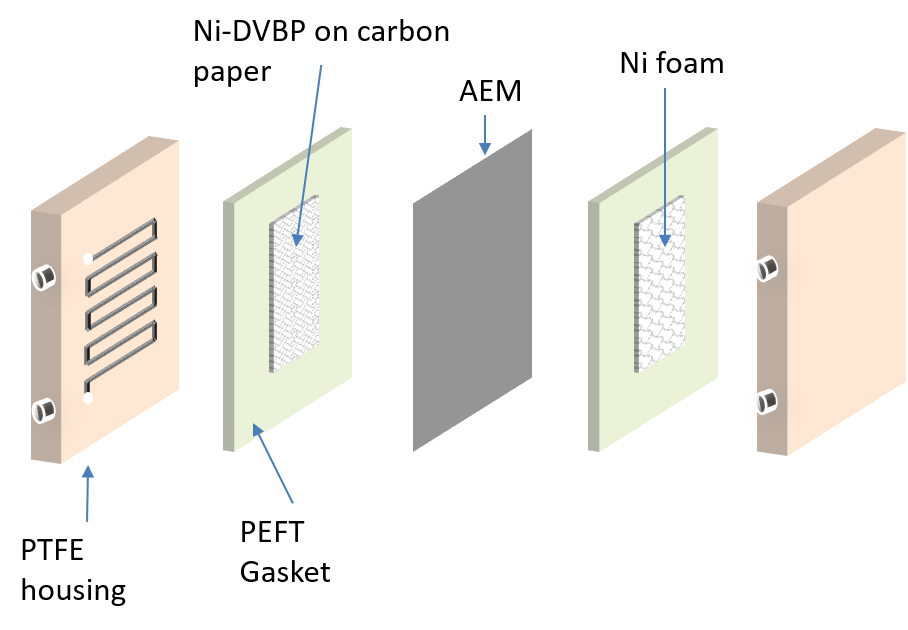


**Supplementary Fig. 1** Diagram of the zero-gap membrane reactor used for flow electrolysis in Method C. The membrane electrode assembly comprises the cathode and anode on either side of the anion exchange membrane (AEM).


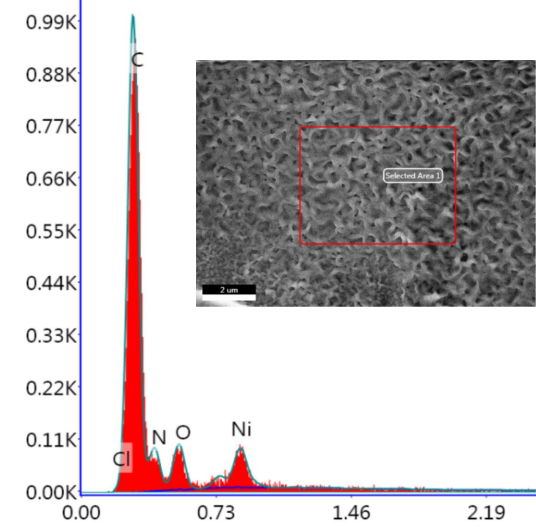


**Supplementary Fig. 2** EDX spectrum at the selected area of Ni-DVBP.


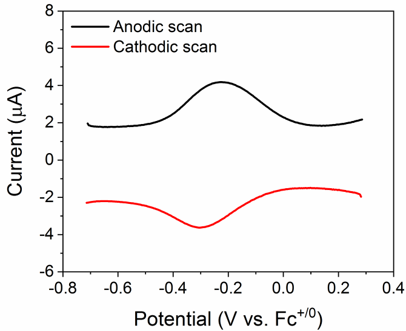


**Supplementary Fig. 3** Square wave spectra of Ni-DVBP in 0.1 M ^n^Bu_4_NPF_6_ MeCN.

The electrode for experiments shown in Supplementary Fig. 3 was prepared via the following procedure. 1 mg of 5,5’-divinyl-2,2’-bipyridine was added in 0.1 mL isopropanol and 10% Benzoyl peroxide (BPO) as radical initiator to prepare stock solution. 2 µL stock solution was loaded on glassy carbon (Φ = 5 mm) and was placed under UV light for 15 min and repeat once. Subsequently, the as prepared electrode was placed in a solution of 100 mM Ni(OTf)_2_ in MeCN overnight. The electrode was washed with MeCN before use.


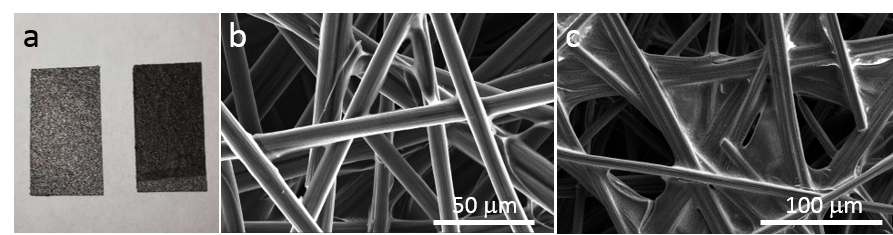


**Supplementary Fig. 4** (a) Photos of carbon paper (left) and DVBP on carbon paper (right). SEM images of (b) carbon paper and (c) DVBP on carbon paper.


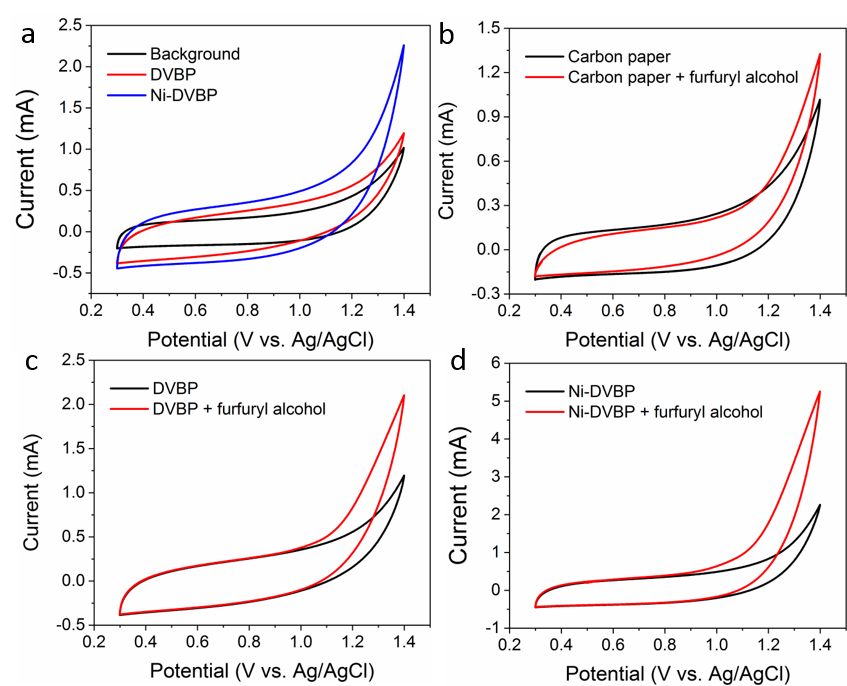


**Supplementary Fig. 5** (a) CV comparison of carbon paper (background), DVBP, and Ni-DVBP in 0.1 M phosphate buffer (KPi, pH 7.03). CV curves of (b) carbon paper, (c) DVBP, and (d) Ni-DVBP with and without 10 mM furfuryl alcohol (FA) in 0.1 M KPi (Electrode area was 1×1 cm^2^).


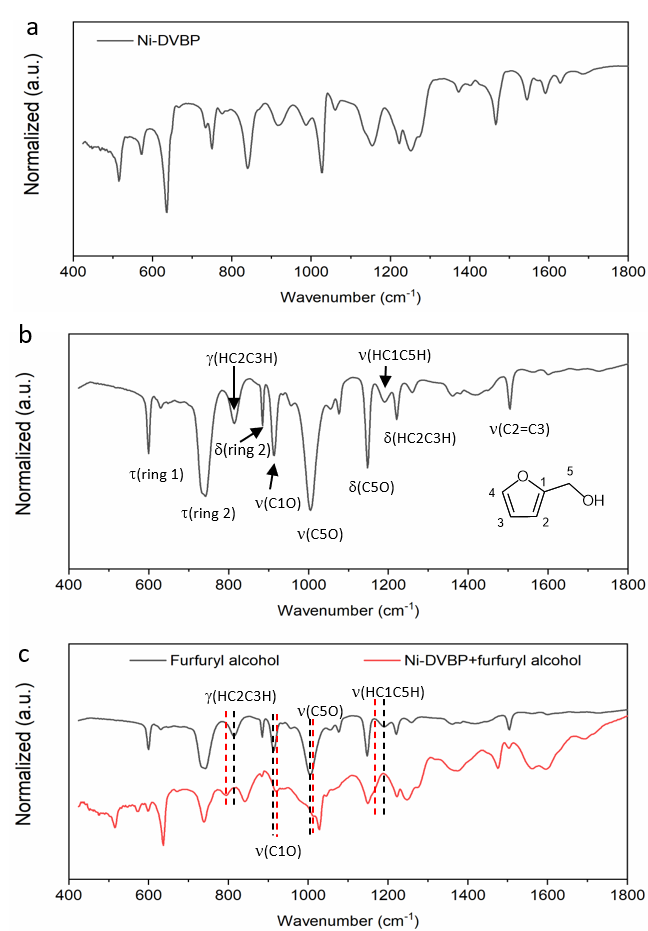


**Supplementary Fig. 6** IR spectra of (a) Ni-DVBP, (b) furfuryl alcohol with the assignments of major features together with the numbered chemical structure of furfuryl alcohol, and (c) Ni-DVBP+furfuryl alcohol with the marked shifts of furfuryl alcohol features. ν, bond stretching; δ, bending; γ, rocking; τ, torsion.


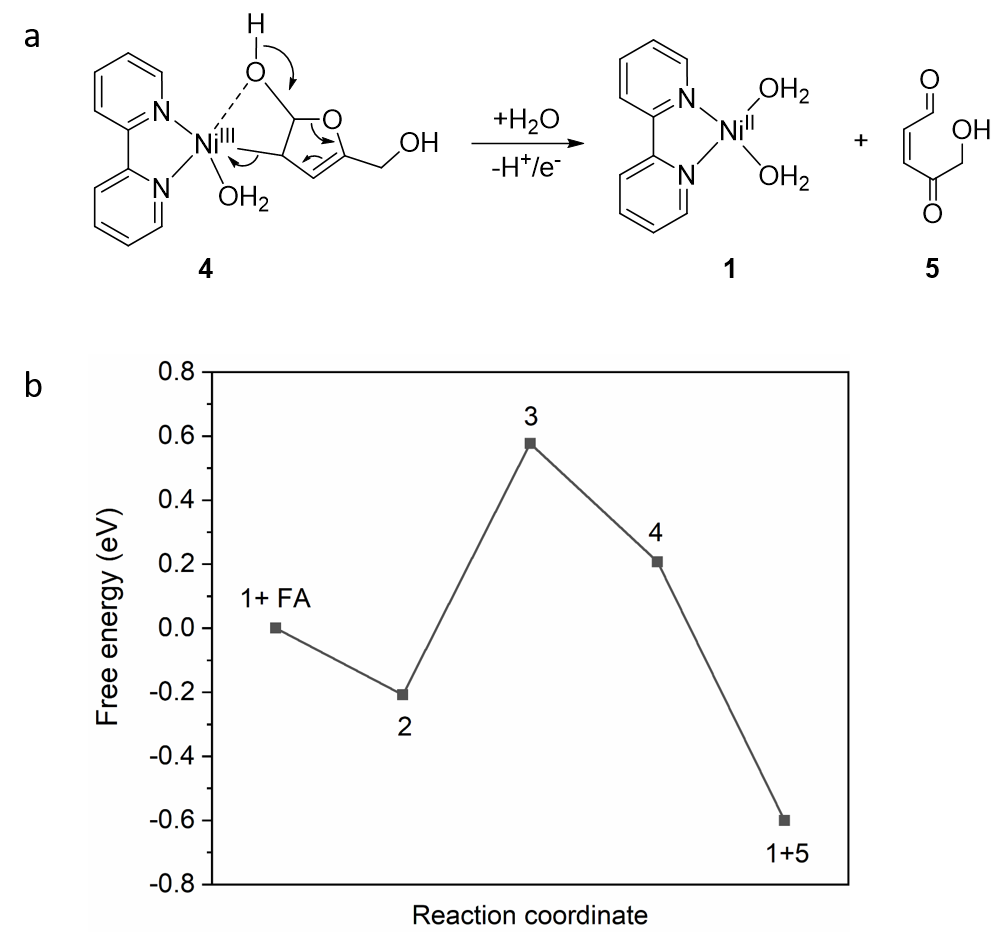


**Supplementary Fig. 7** (a) Proposed electron pushing process for the generation of intermediate **5** from **4**. (b) DFT-calculated energy profile of Mechanism 1. FA: Furfuryl alcohol.





**Supplementary Fig. 8** DFT-calculated energy profile of Mechanism 2.





**Supplementary Fig. 9** Chronoamperometric curves of furfuryl alcohol oxidation on Ni-DVBP or carbon paper in 0.1 M KPi at different applied potentials.

**Supplementary Table 1.** Summary of furfuryl alcohol oxidation on Ni-DVBP vs carbon paper (CP) in 0.1 M KPi at different applied potentials.

| Potential (V vs Ag/AgCl) | FA conversion (%) | | HPO selectivity (%) | | HPO Yields (%) | |
| --- | --- | --- | --- | --- | --- | --- |
|  | Ni-DVBP | CP | Ni-DVBP | CP | Ni-DVBP | CP |
| 1.1 | >99 | ~0 | 79 | N/A | 79 | N/A |
| 1.2 | >99 | ~0 | 87 | N/A | 87 | N/A |
| 1.3 | >99 | 38 | 92 | 28 | 92 | 11 |
| 1.4 | >99 | 42 | 94 | 33 | 94 | 14 |





**Supplementary Fig. 10** Hydropyranone (HPO) yields in 0.1 M phosphate buffer utilizing Ni-DVBP and carbon paper as working electrodes at different potentials.

**^1^H NMR spectra**


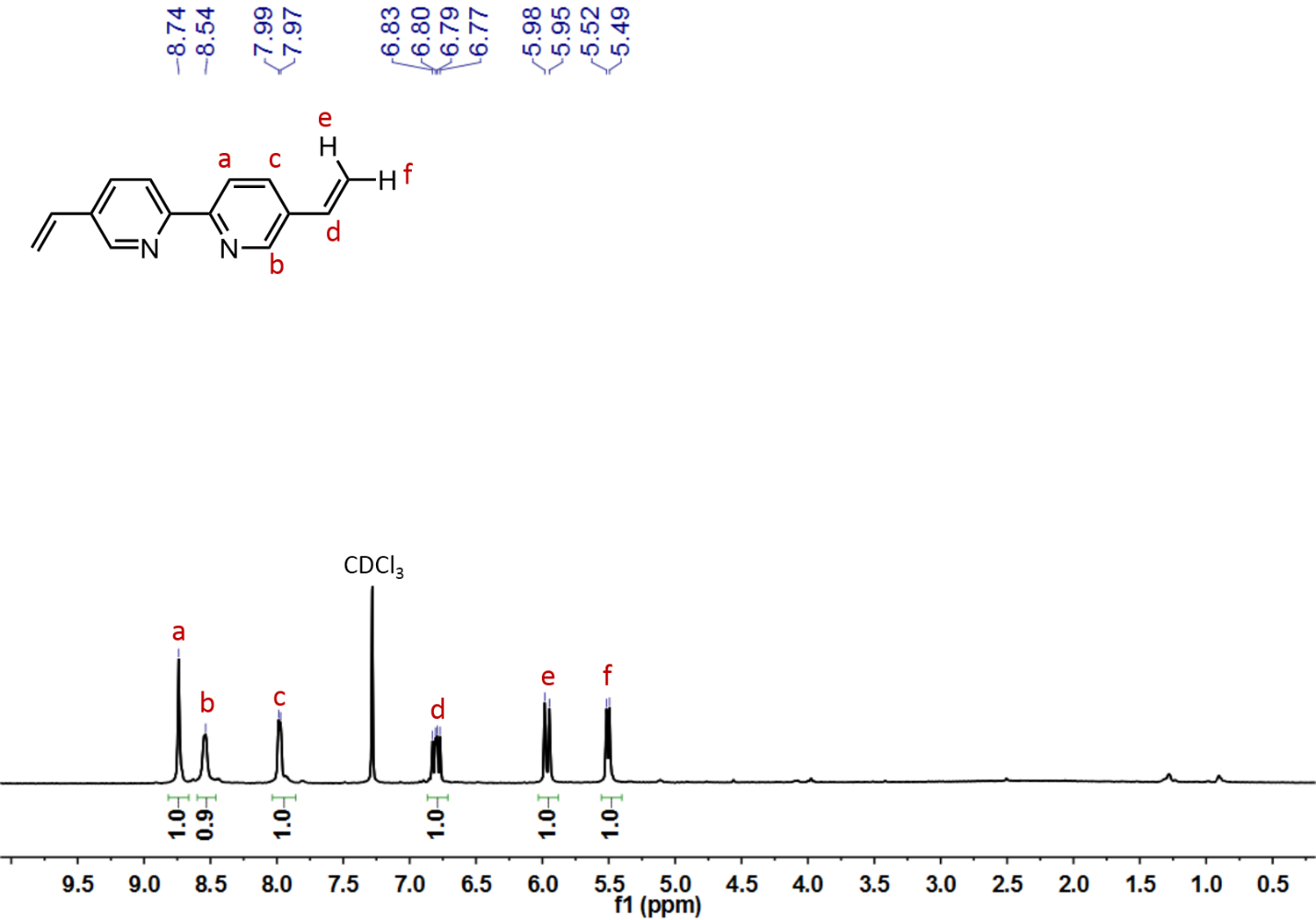


**5,5'-divinyl-2,2'-bipyridine (dvbpy):** ^1^H NMR (500 MHz, CDCl_3_) δ 8.74 (s, 1H), 8.54 (s, 1H), 7.98 (d, *J* = 7.6 Hz, 1H), 6.80 (dd, *J* = 17.3, 11.1 Hz, 1H), 5.96 (d, *J* = 17.7 Hz, 1H), 5.51 (d, *J* = 10.9 Hz, 1H).

**6-Hydroxy-2H-pyran-3(6H)-one (2a):** Followed Method B from 1a (49 mg, 0.50 mmol) and purified using preparative chromatography to give 52.5 mg (92% yield) of 2a as a colorless oil. ^1^H NMR (400 MHz, CDCl_3_) δ 6.90 (dd, *J* = 10.2, 3.0 Hz, 1H), 6.10 (d, *J* = 10.3 Hz, 1H), 5.57 (d, *J* = 3.0 Hz, 1H), 4.51 (d, *J* = 16.9 Hz, 1H), 4.07 (d, *J* = 16.9 Hz, 1H). ^13^C NMR (101 MHz, CDCl_3_) δ 193.64, 144.83, 126.89, 87.20, 65.60.


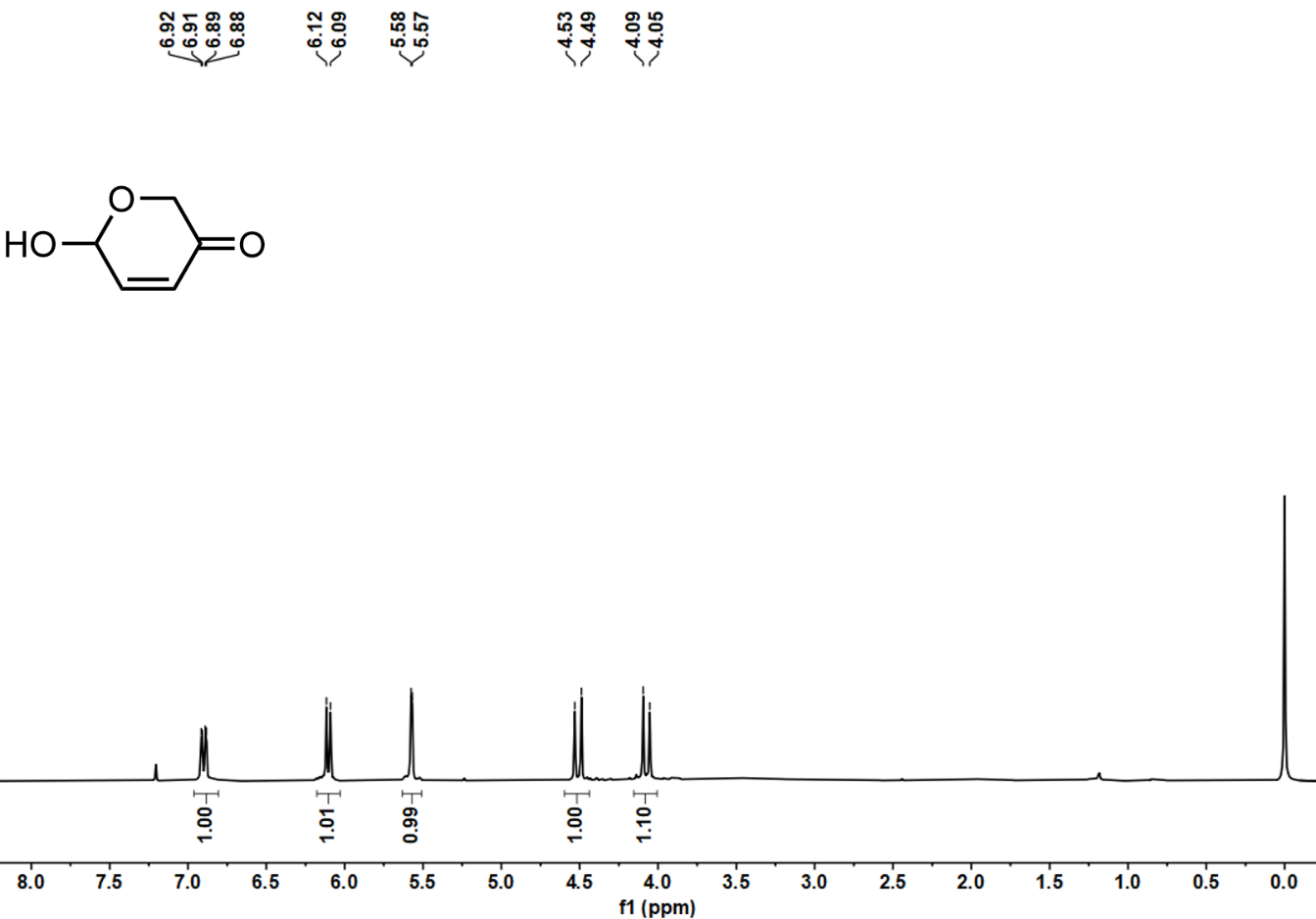


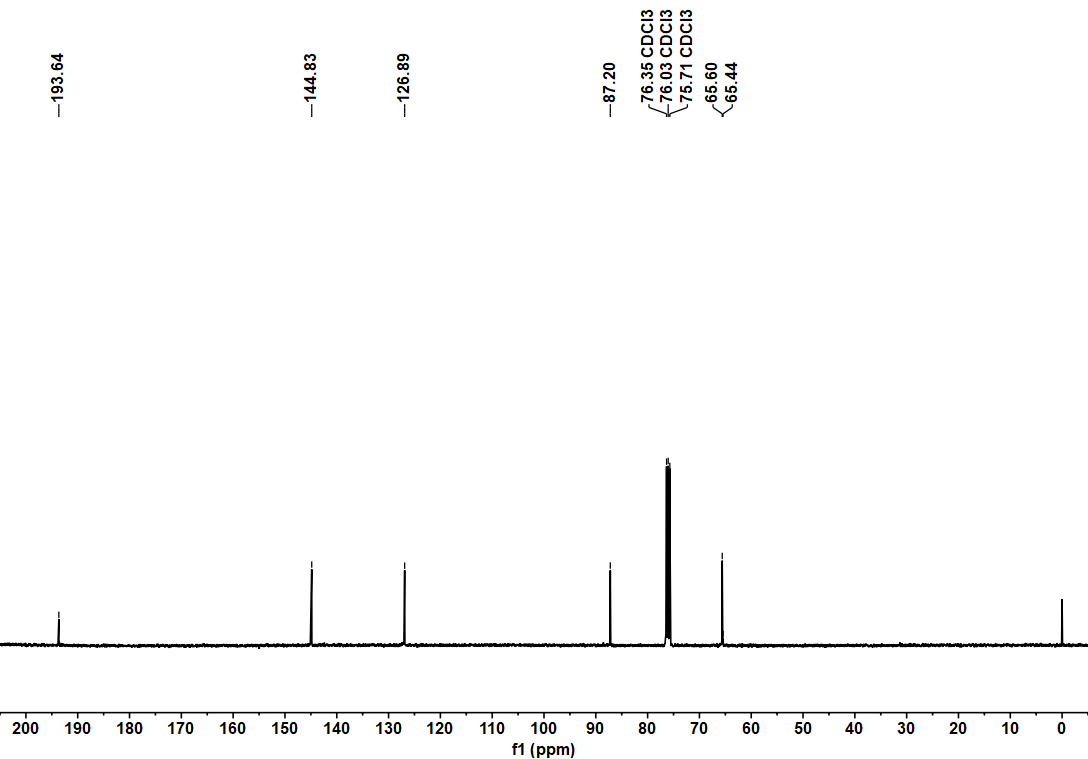


**6-Hydroxy-2-methyl-2H-pyran-3(6H)-one (2b):** Followed Method B from 1b (56 mg, 0.50 mmol) and purified using preparative chromatography to give 57.9 mg (93% yield) of 2b as a colorless oil. ^1^H NMR (400 MHz, ) mixture of isomers: δ 6.97 – 6.75 (m, 1H), 6.06 (dd, *J* = 19.4, 10.2 Hz, 1H), 5.70 – 5.52 (m, 1H), 4.72 – 4.14 (m, 1 H), 3.22-2.98 (s, 1H), 1.40 (dd, *J* = 6.7, 1.6 Hz, 1H), 1.33 (dd, *J* = 6.7, 1.6 Hz, 2H). ^13^C NMR (101 MHz, CDCl_3_) δ 196.66, 147.55, 144.21, 128.81, 127.72, 90.88, 87.67, 78.73, 74.00, 32.66, 31.63, 18.39, 18.24, 13.84, 13.80.


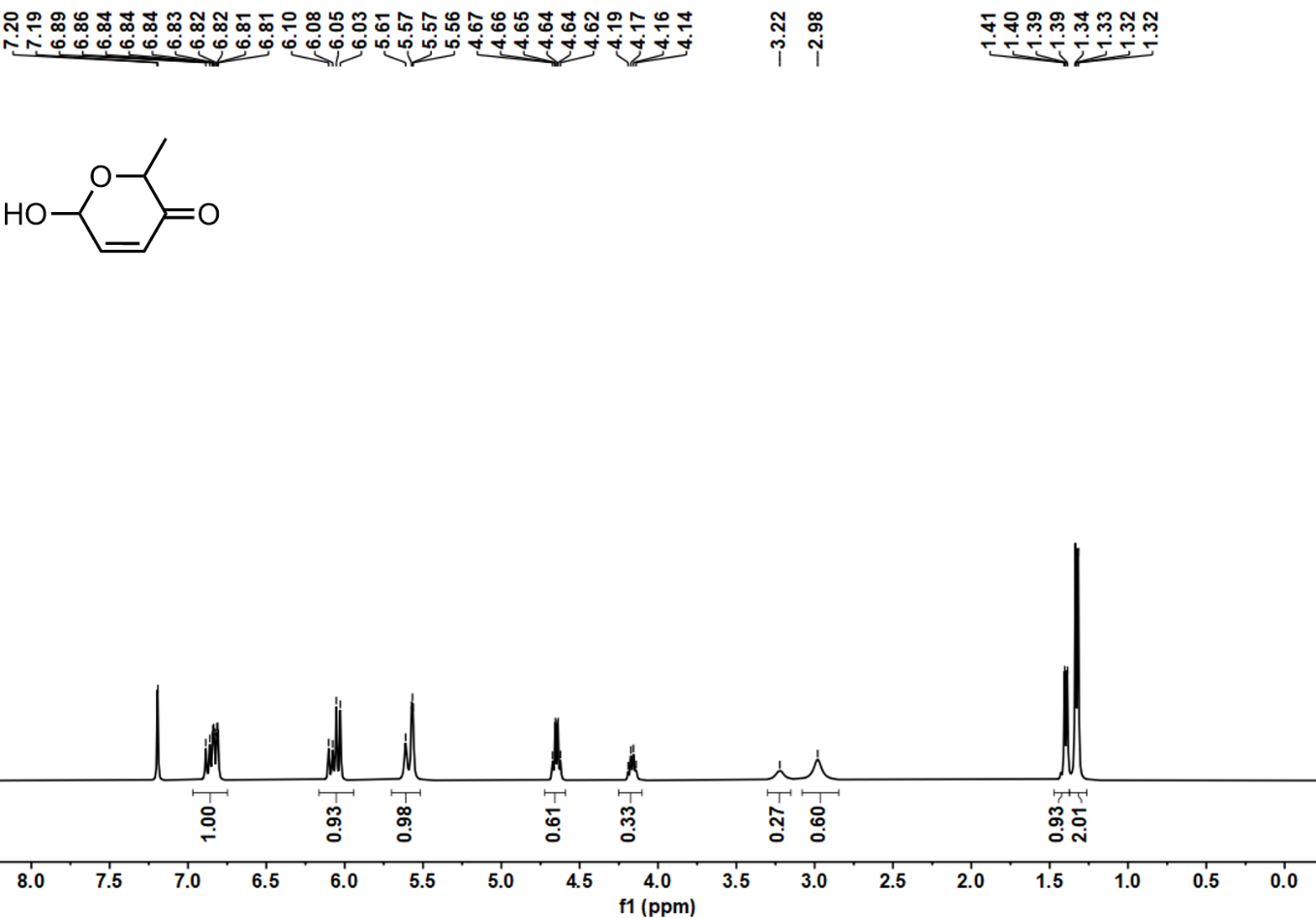


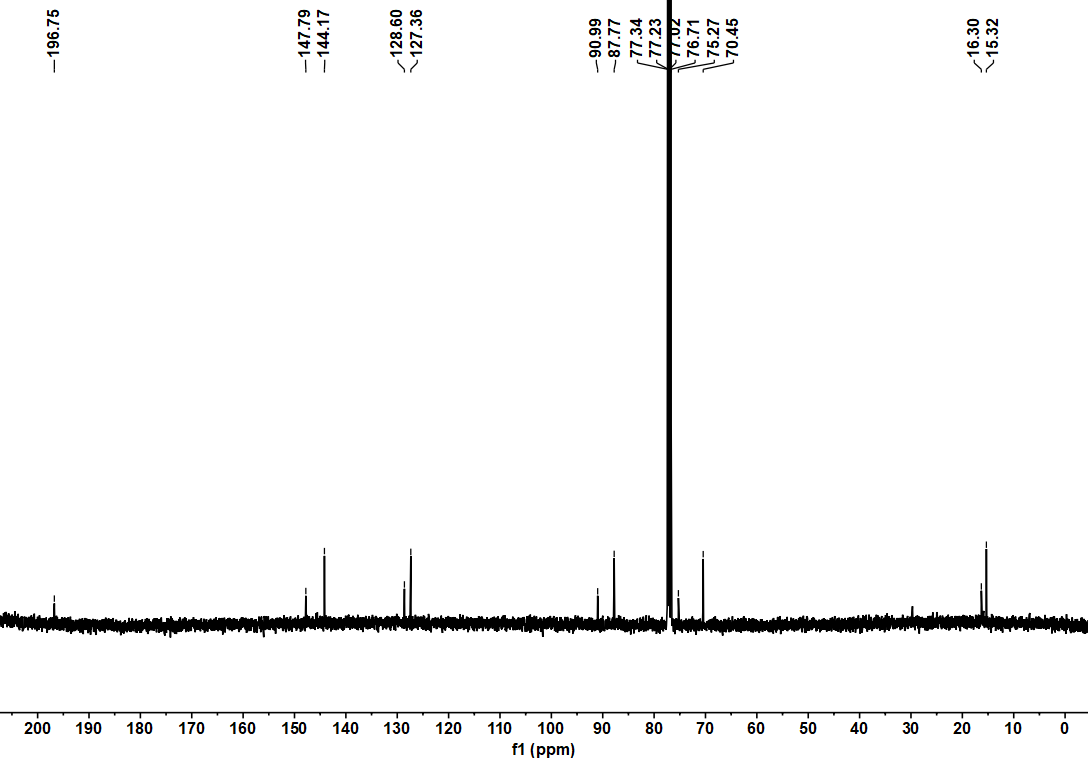


**6-Hydroxy-2,2-dimethyl-2H-pyran-3(6H)-one (2c):** Followed Method B from 1c (63 mg, 0.50 mmol) and purified using preparative chromatography to give 61.8 mg (87% yield) of 2c as a colorless oil. ^1^H NMR (400 MHz, CDCl_3_) δ 6.82 (dt, *J* = 10.3, 2.1 Hz, 1H), 6.01 (d, *J* = 10.3 Hz, 1H), 5.63 (s, 1H), 3.32 (s, 1H), 1.38 (dd, *J* = 39.1, 1.8 Hz, 7H). ^13^C NMR (101 MHz, CDCl_3_) δ 198.87, 145.66, 126.49, 87.90, 79.44, 26.56, 23.78.


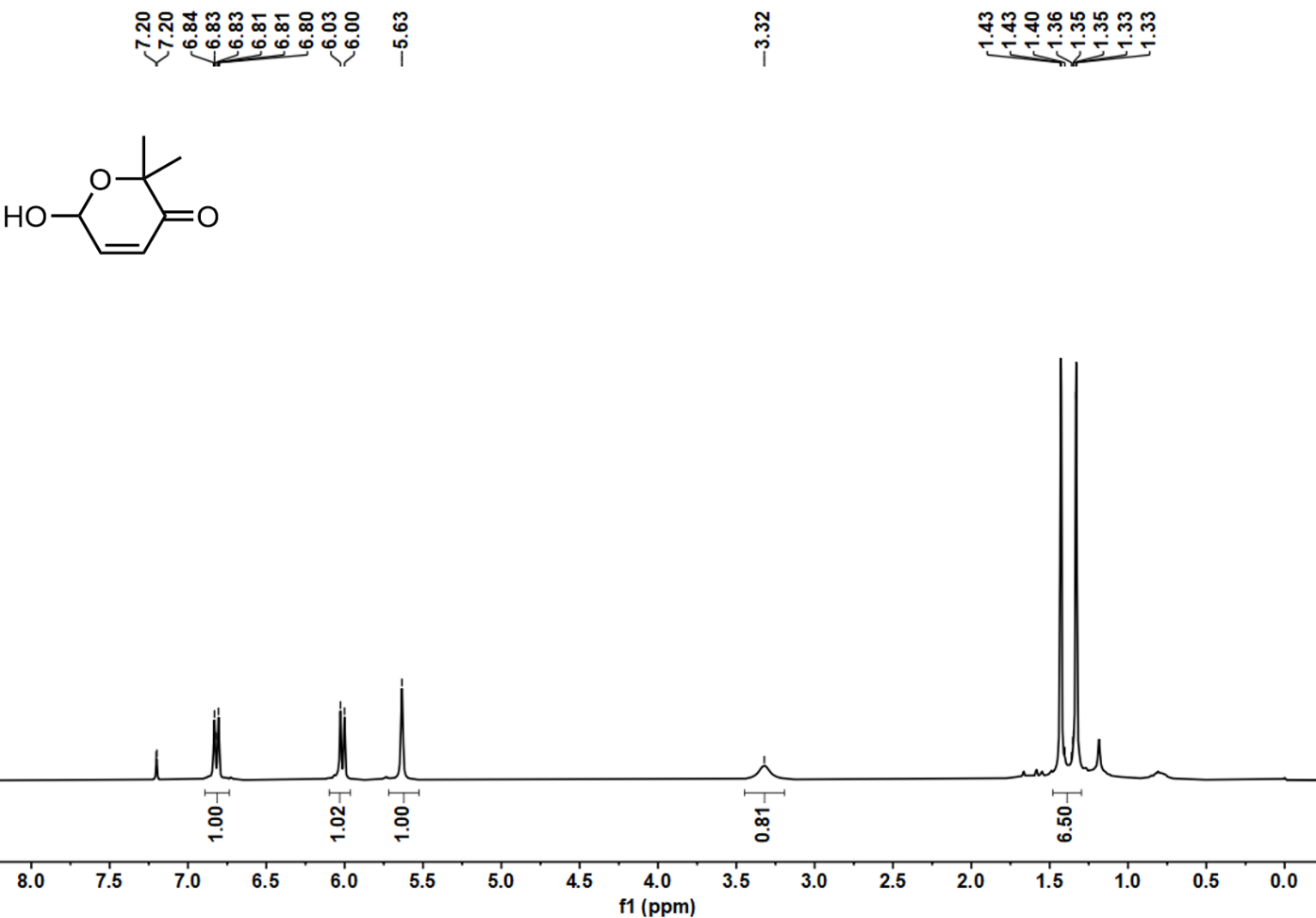


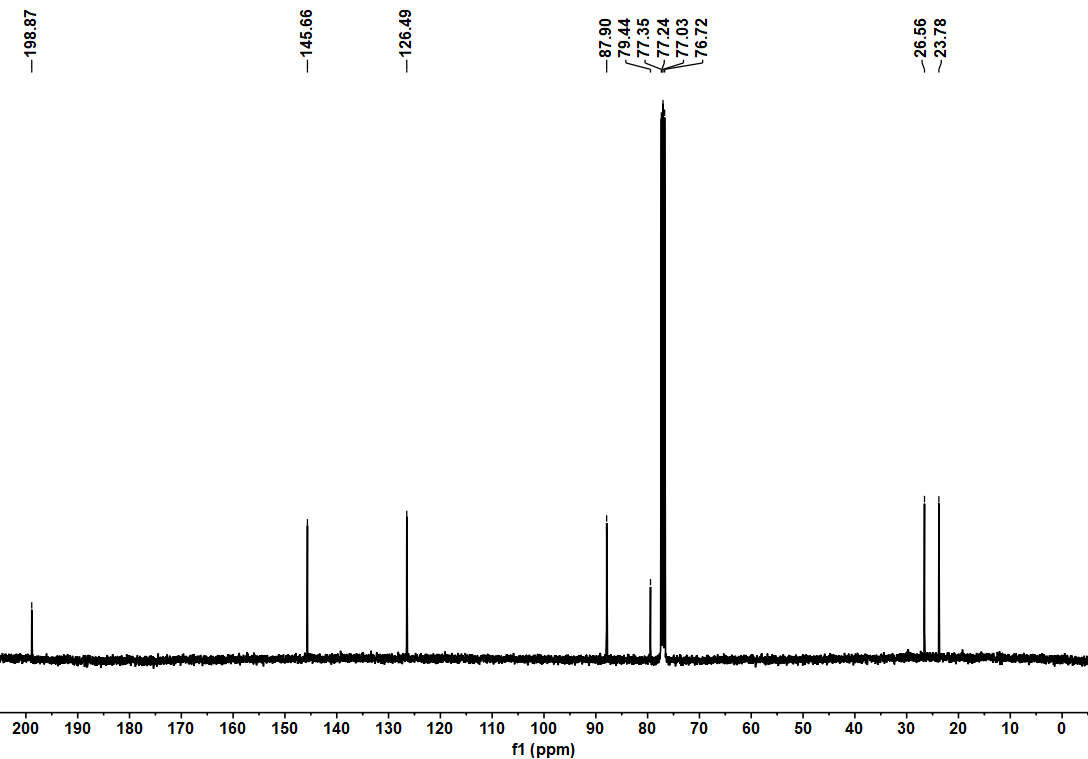


**6-Hydroxy-2-propyl-2H-pyran-3(6H)-one (2d):** Followed Method B from 1d (70 mg, 0.50 mmol) and purified using preparative chromatography to give 70.6 mg (90% yield) of 2d as a colorless oil.  ^1^H NMR (400 MHz, CDCl_3_) mixture of isomers: δ 6.95 – 6.71 (m, 1H), 6.06 (dd, *J* = 16.7, 10.3 Hz, 1H), 5.58 (d, *J* = 3.7 Hz, 1H), 4.50 (dd, *J* = 8.3, 3.8 Hz, 0.7H), 4.02 (dd, *J* = 8.5, 4.0 Hz, 0.3 H), 3.04 (s, 1H), 1.84 (tt, *J* = 13.5, 5.3 Hz, 1H), 1.64 (tt, *J* = 14.3, 8.5 Hz, 1H), 1.52 – 1.29 (m, 2H), 0.87 (td, *J* = 7.4, 1.8 Hz, 3H). ^13^C NMR (101 MHz, CDCl_3_) δ 196.75, 147.79, 144.17, 128.60, 127.36, 90.99, 87.77, 75.27, 70.45, 16.30, 15.32.


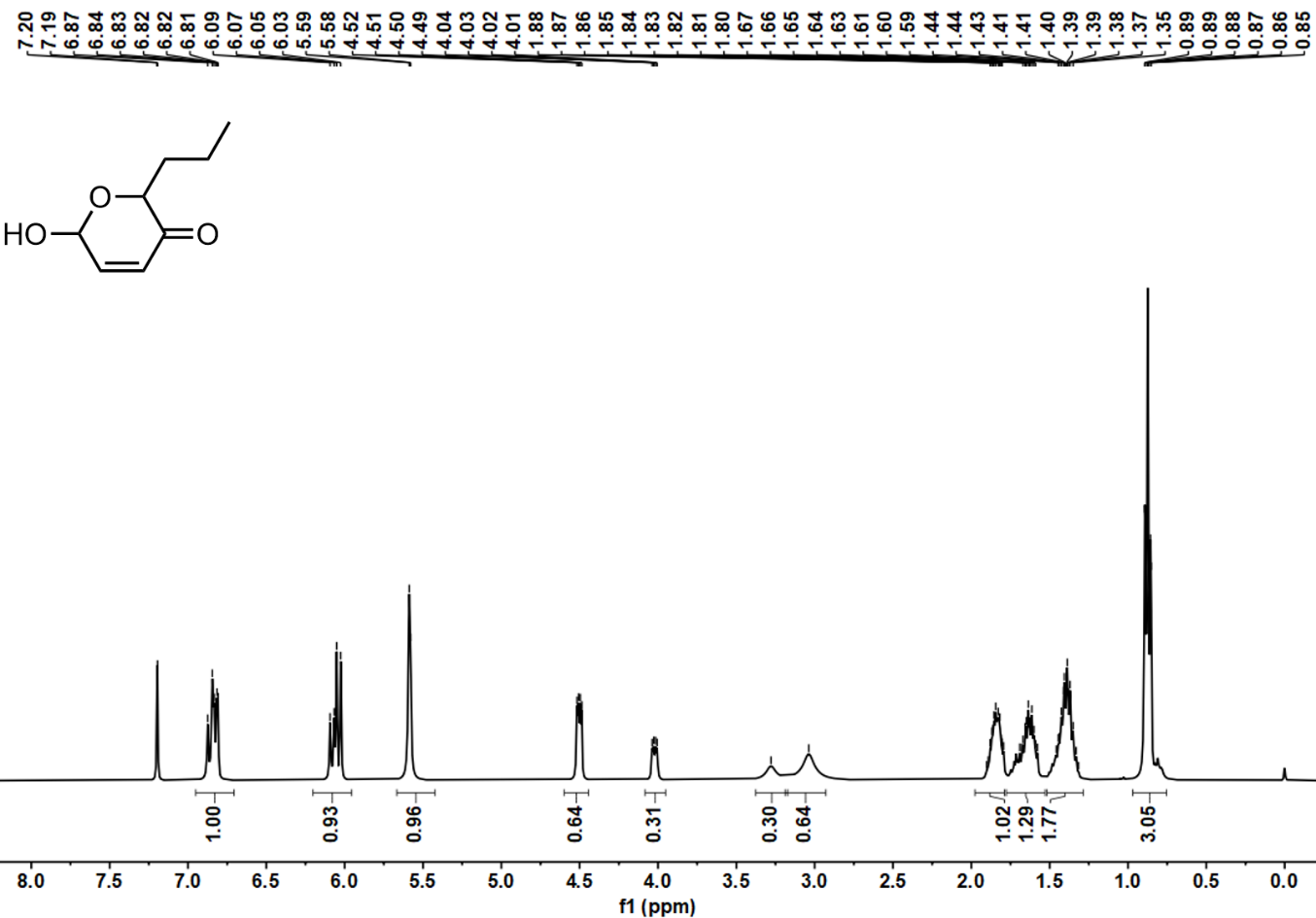


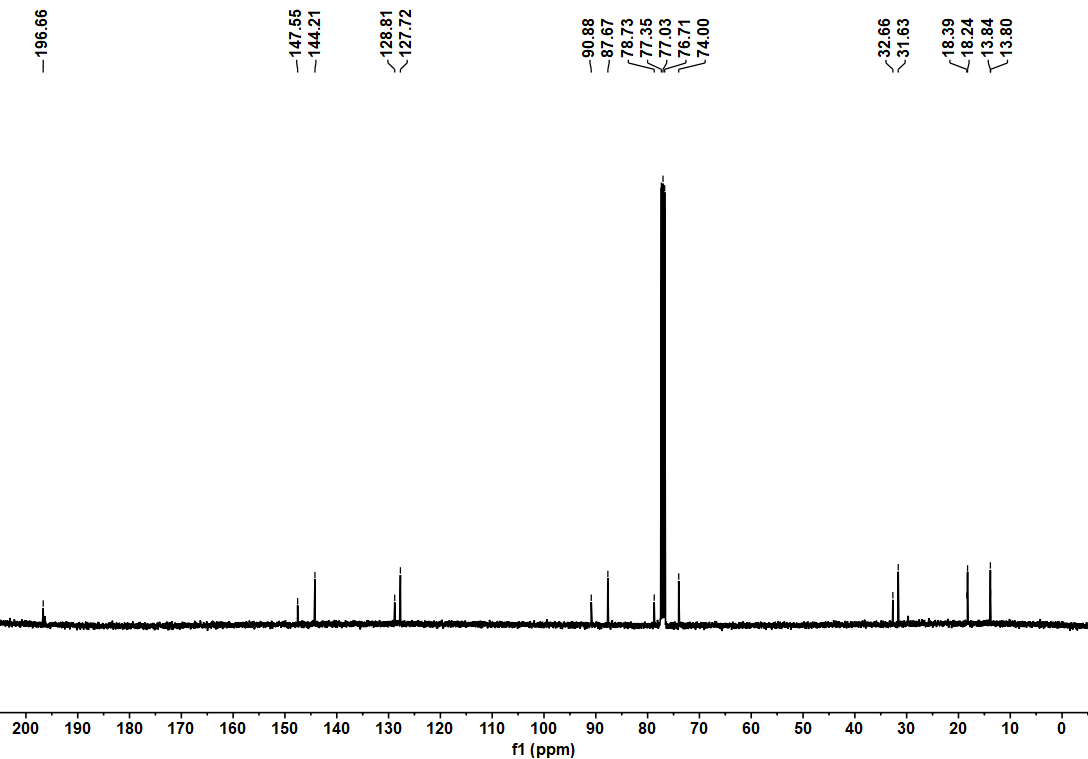


**1-(Furan-2-yl)-2-phenylethan-1-ol (2e)** Followed Method B from 1e (94 mg, 0.50 mmol) and purified using preparative chromatography to give 79 mg (78% yield) of 2e as a colorless oil.  ^1^H NMR (400 MHz, CDCl_3_) mixture of isomers: δ 7.28 (tt, *J* = 14.0, 7.3 Hz, 5H), 6.95 – 6.77 (m, 1H), 6.14 (dd, *J* = 19.7, 10.2 Hz, 1H), 5.54 – 5.38 (m, 1H), 4.84 (dd, *J* = 9.4, 3.2 Hz, 0.7H), 4.29 (dd, J = 9.7, 3.1 Hz, 0.3H), 3.67 (s, 0.3H), 3.39 (ddd, *J* = 13.4, 9.7, 3.3 Hz, 1.7H), 2.93 (ddd, *J* = 35.2, 14.8, 9.5 Hz, 1H).


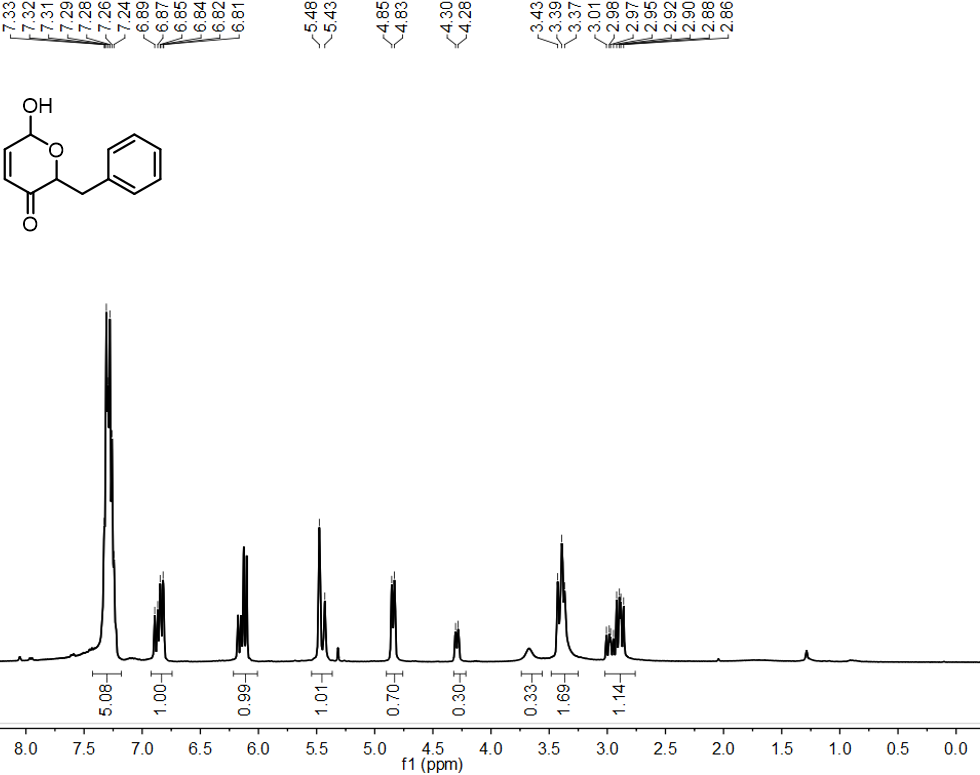


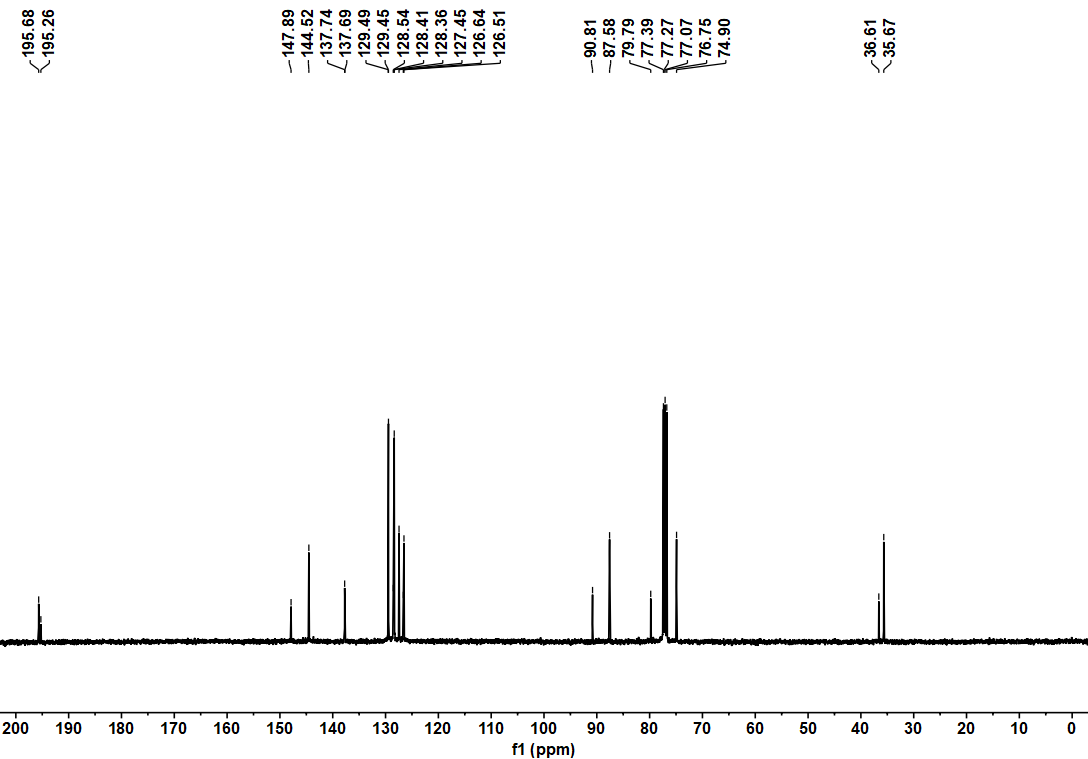


**6-Hydroxy-2-phenethyl-2H-pyran-3(6H)-one (2f)** Followed Method B from 1f (100 mg, 0.50 mmol) and purified using preparative chromatography to give 89 mg (82% yield) of 2f as a colorless oil.  ^1^H NMR (400 MHz, CDCl_3_) mixture of isomers: δ 7.17 (dp, *J* = 22.1, 7.3 Hz, 5H), 6.89 – 6.79 (m, 1H), 6.06 (dd, *J* = 17.1, 10.0 Hz, 1H), 5.63 – 5.56 (m, 1H), 4.48 (dd, *J* = 8.3, 3.7 Hz, 0.6 H), 3.97 (dd, *J* = 9.0, 3.8 Hz, 0.4 H), 3.36 (s, 0.3 H), 3.04 (s, 0.7 H), 2.86 – 2.65 (m, 2H), 2.20 (dddd, *J* = 13.8, 10.1, 7.3, 4.0 Hz, 1H), 1.97 (dddd, *J* = 31.9, 14.5, 9.1, 5.2 Hz, 1H), 1.61 (s, 0.8H), 1.16 (m, 0.5H).


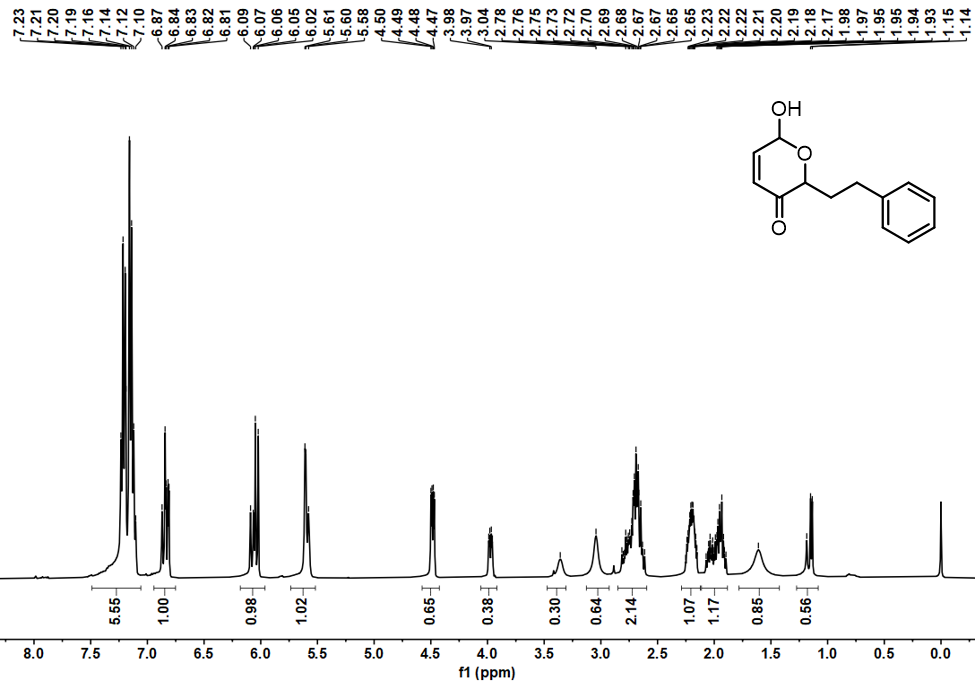


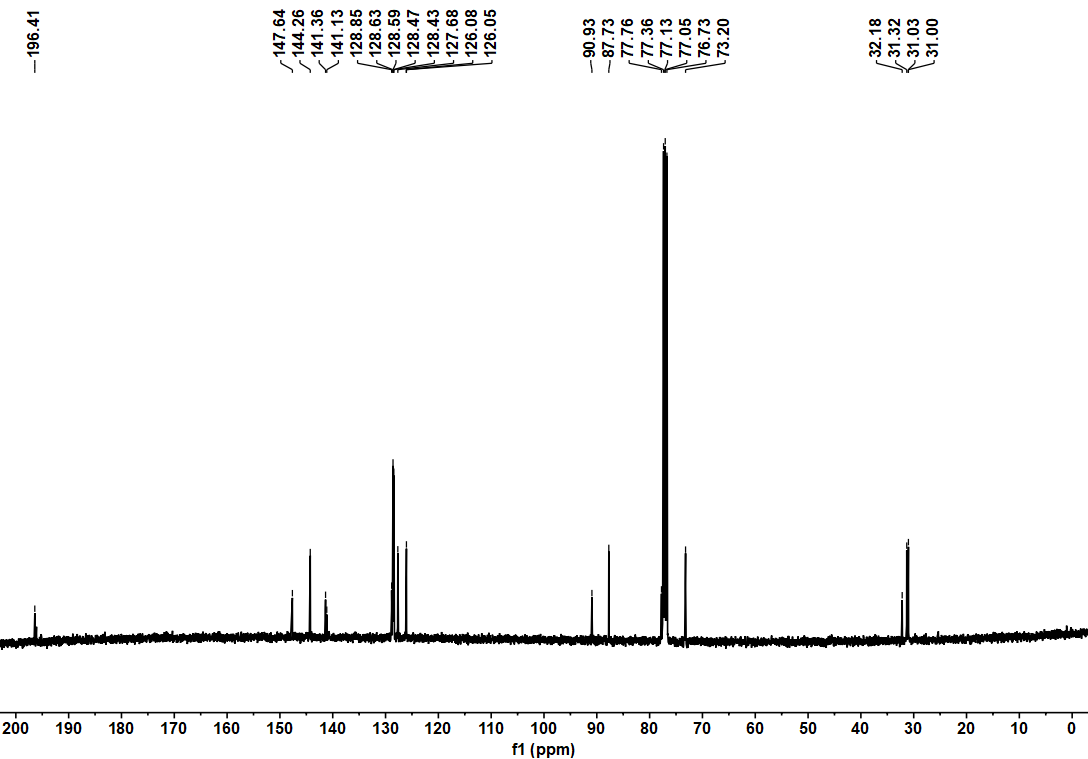


**2-Benzyl-6-hydroxy-2H-pyran-3(6H)-one (2g)** Followed Method B from 1g (87 mg, 0.50 mmol) and purified using preparative chromatography to give 86 mg (91% yield) of 2g as a colorless oil.  ^1^H NMR (400 MHz, CDCl_3_) mixture of isomers: δ 7.29 (qd, *J* = 9.2, 8.3, 4.3 Hz, 5H), 6.96 – 6.84 (m, 1H), 6.16 (dd, *J* = 21.3, 10.3 Hz, 1H), 5.64 (d, *J* = 3.3 Hz, 1H), 5.51 (s, 0.8H), 5.02 (s, 0.2H), 3.48 (bs, 1H).


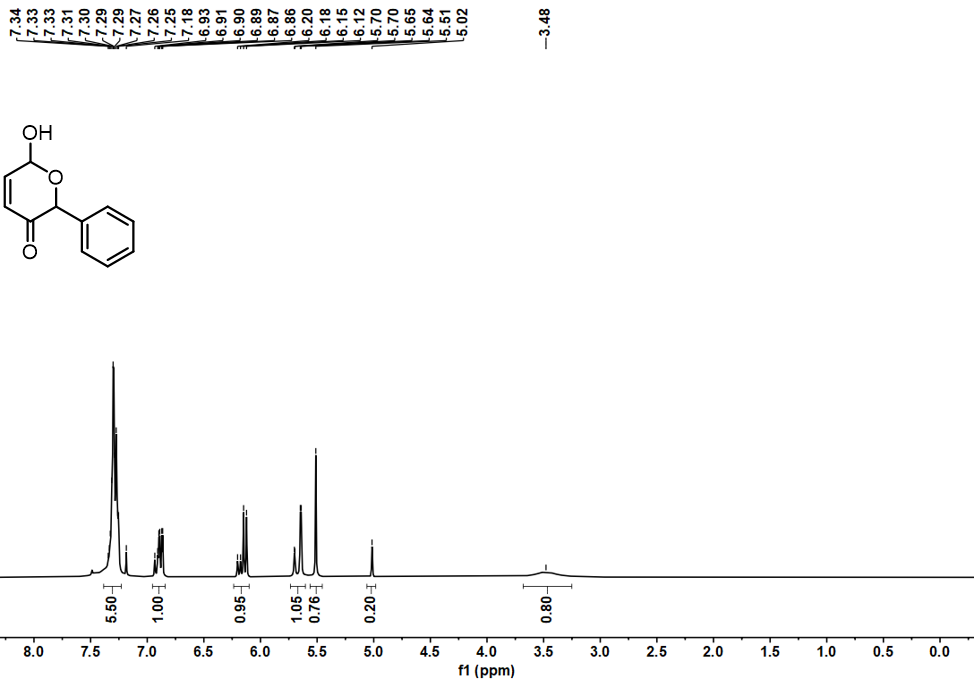


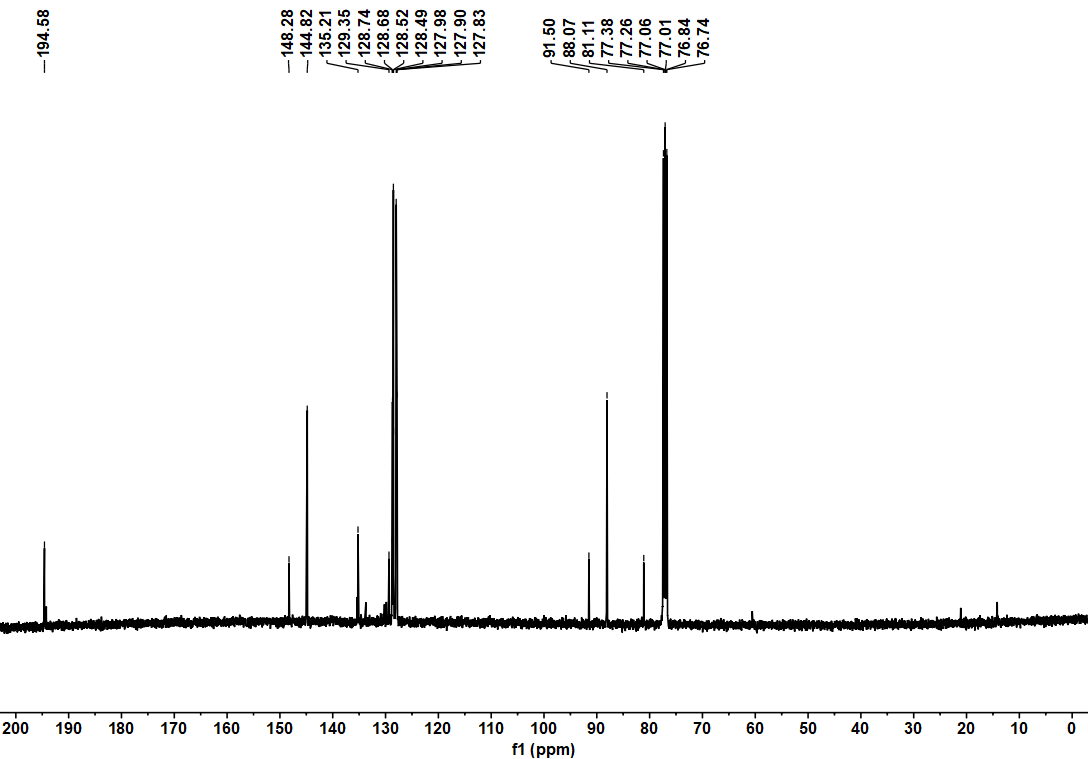


**6-Hydroxy-2-(p-tolyl)-2H-pyran-3(6H)-one (2h)** Followed Method B from 1h (94 mg, 0.50 mmol) and purified using preparative chromatography to give 91 mg (89% yield) of 2h as a colorless oil.  ^1^H NMR (400 MHz, CDCl_3_) mixture of isomers: δ 7.25 (dd, *J* = 20.1, 6.3 Hz, 4H), 6.97 (ddd, *J* = 15.8, 10.2, 2.4 Hz, 1H), 6.25 (dd, *J* = 20.0, 9.4 Hz, 1H), 5.85 – 5.65 (m, 1H), 5.57 (s, 0.7H), 5.06 (s, 0.2H), 3.77 (bs, 1H), 2.38 (s, 3H).


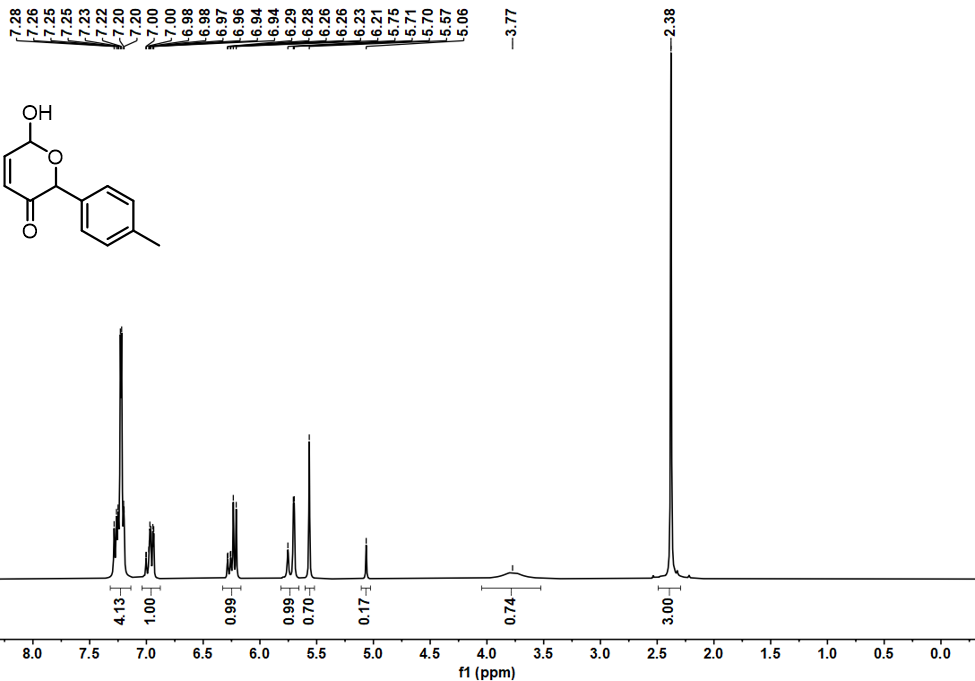


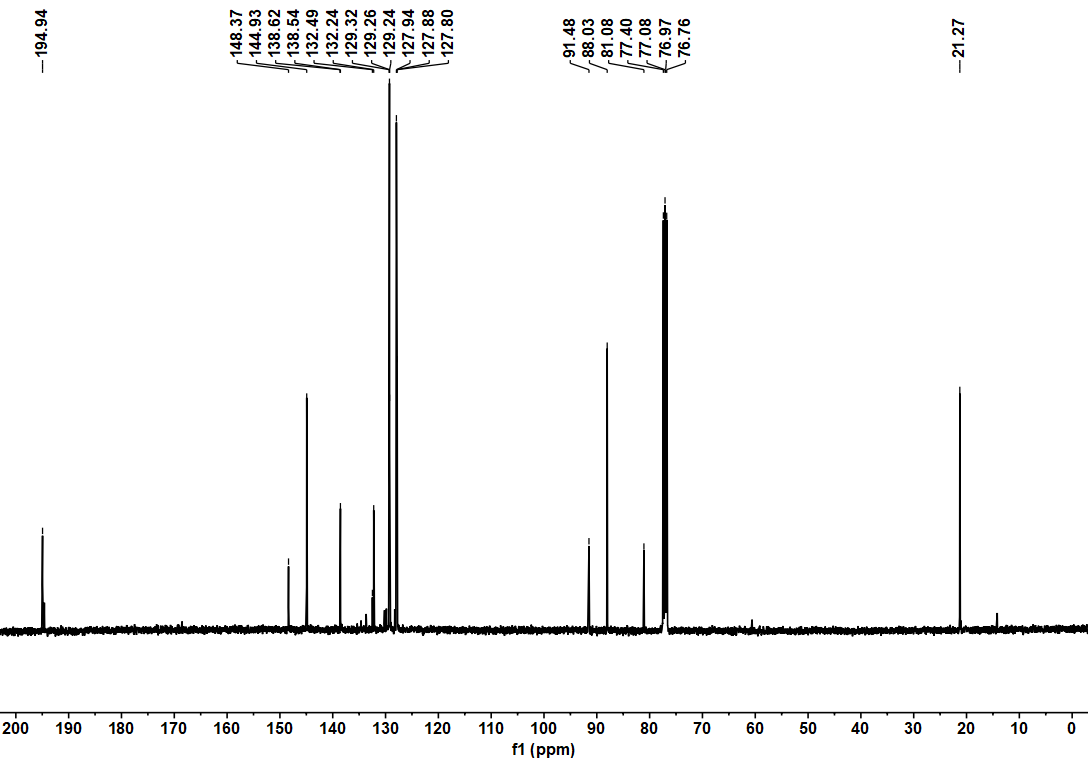


**2-(4-Fluorophenyl)-6-hydroxy-2H-pyran-3(6H)-one (2i)** Followed Method B from 1i (96 mg, 0.50 mmol) and purified using preparative chromatography to give 79 mg (76% yield) of 2i as a colorless oil.  ^1^H NMR (400 MHz, CDCl_3_) mixture of isomers: δ 7.26 (dt, *J* = 8.5, 5.7 Hz, 2H), 7.05 – 6.93 (m, 2H), 6.91 (d, *J* = 3.4 Hz, 1H), 6.15 (d, *J* = 10.2 Hz, 1H), 5.76-5.69 (0H), 5.52 (s, 0.8H), 5.03 (s, 0.2H), 3.40 (s, 0.3H), 3.22 (s, 0.7H).


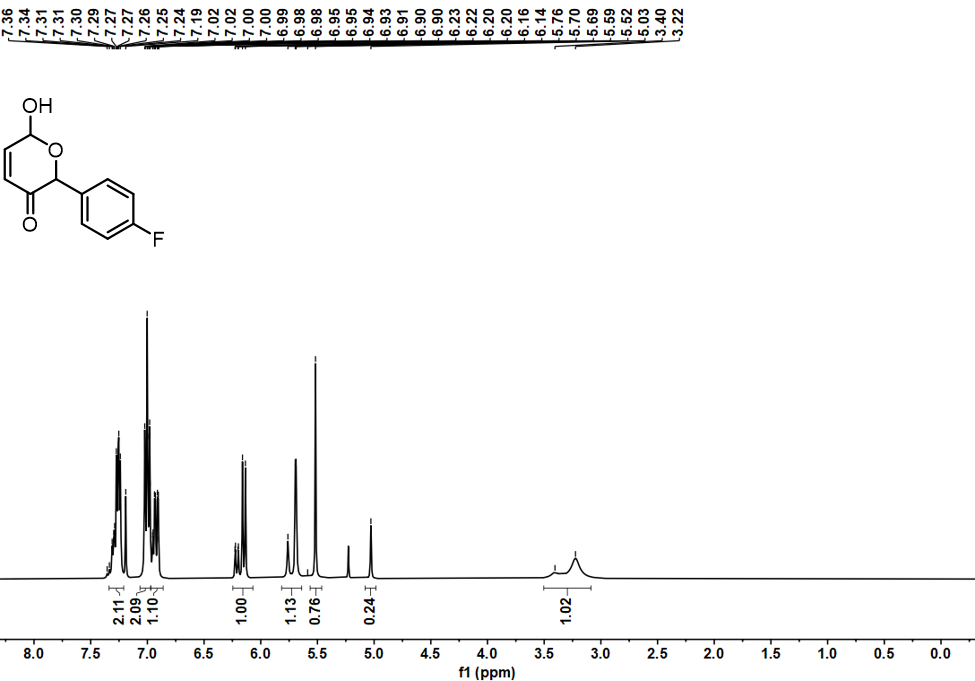


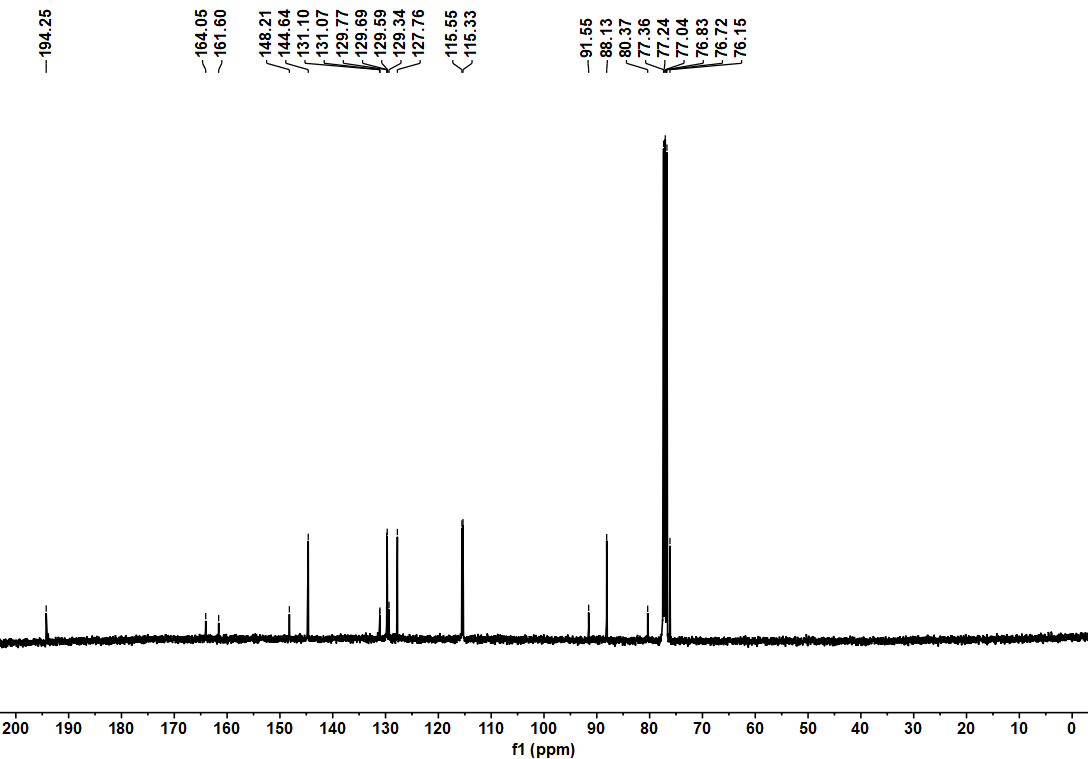


**6-Hydroxy-6-methyl-2H-pyran-3(6H)-one (2j):** Followed Method B from 1j (56 mg, 0.50 mmol) and purified using preparative chromatography to give 52.6 mg (82% yield) of 2j as a colorless oil.  ^1^H NMR (400 MHz, CDCl_3_) δ 6.87 (d, *J* = 10.3 Hz, 1H), 6.08 (d, *J* = 10.3 Hz, 1H), 4.55 (t, *J* = 15.0 Hz, 1H), 4.13 (d, *J* = 16.9 Hz, 1H), 1.65 (s, 3H). ^13^C NMR (101 MHz, CDCl_3_) δ 194.76, 148.72, 126.58, 92.84, 66.59, 28.00.


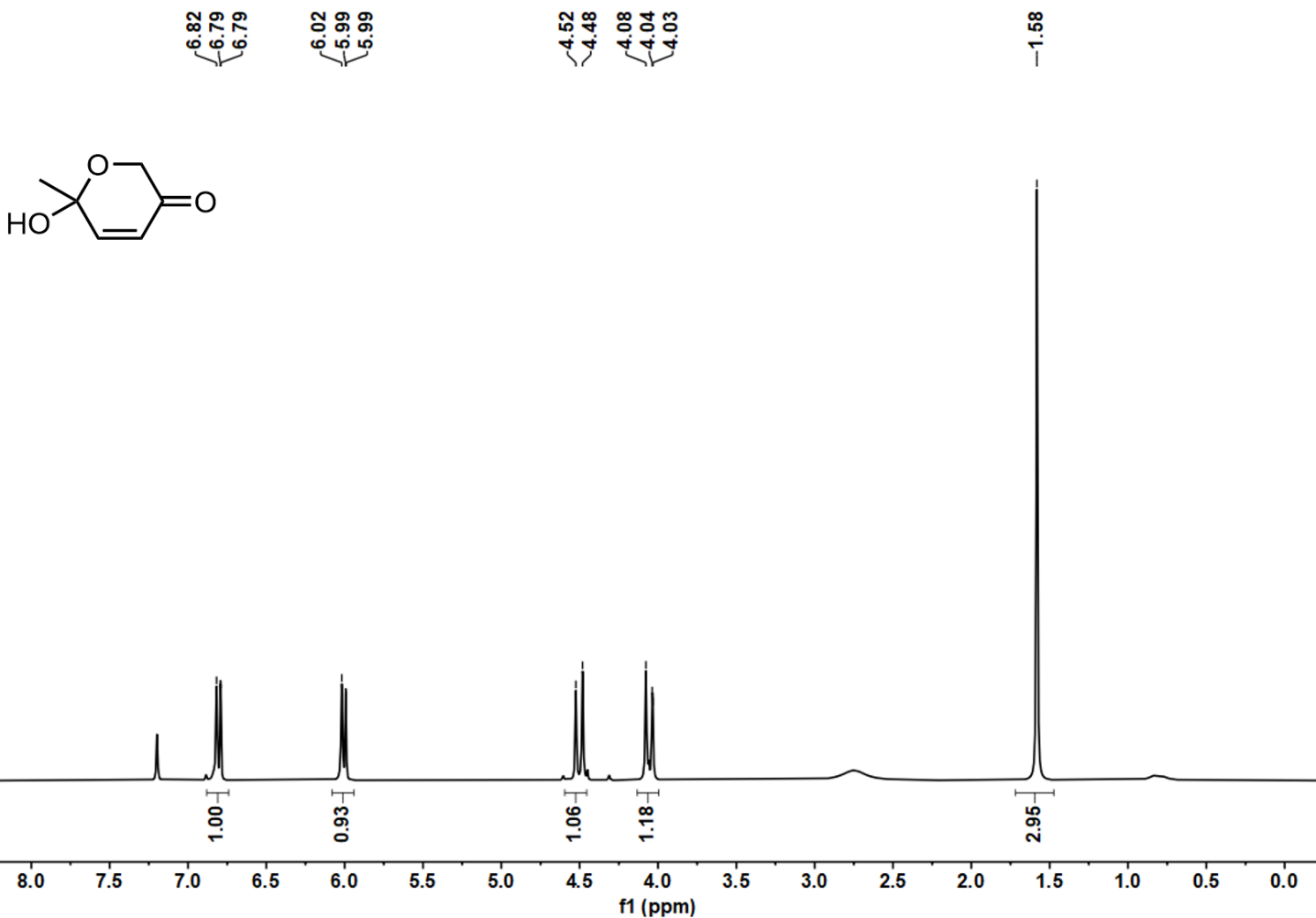


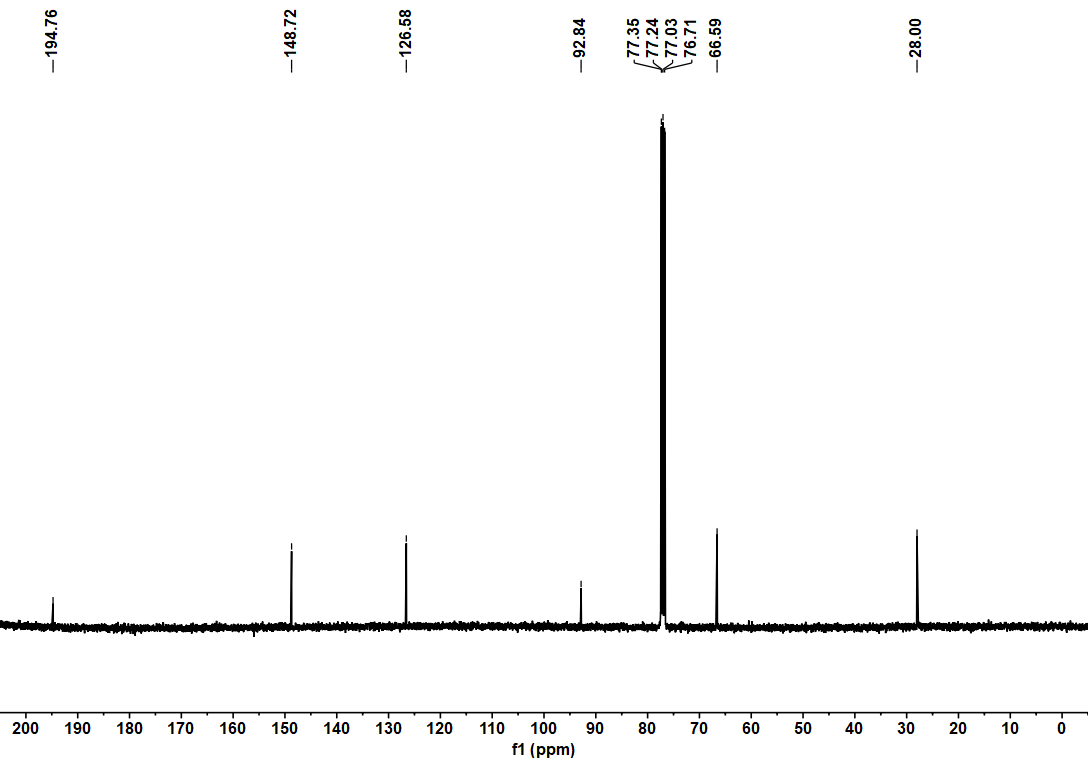


**6-(4-chlorophenyl)-6-hydroxy-2H-pyran-3(6H)-one (2k)** Followed Method B from 1k (105 mg, 0.50 mmol) and purified using preparative chromatography to give 94 mg (84% yield) of 2j as a white solid. ^1^H NMR (400 MHz, CDCl_3_) δ 7.86 (d, *J* = 8.6 Hz, 2H), 7.58 (d, *J* = 8.6 Hz, 2H), 7.47 (d, *J* = 8.6 Hz, 2H), 7.39 (d, *J* = 8.6 Hz, 2H), 6.96 (d, *J* = 12.0 Hz, 1H), 6.88 (d, *J* = 10.3 Hz, 1H), 6.57 (d, *J* = 12.0 Hz, 1H), 6.10 (d, *J* = 10.3 Hz, 1H), 4.72 (d, *J* = 16.8 Hz, 1H), 4.39 (s, 1H), 4.27 (d, *J* = 16.8 Hz, 1H), 3.19 (s, 1H), 2.97 (s, 1H). ^13^C NMR (101 MHz, CDCl_3_) δ 199.86, 193.90, 191.95, 148.23, 140.65, 140.20, 138.24, 135.39, 133.80, 131.32, 130.01, 128.91, 127.30, 126.17, 93.77, 68.37, 66.76, 29.71.


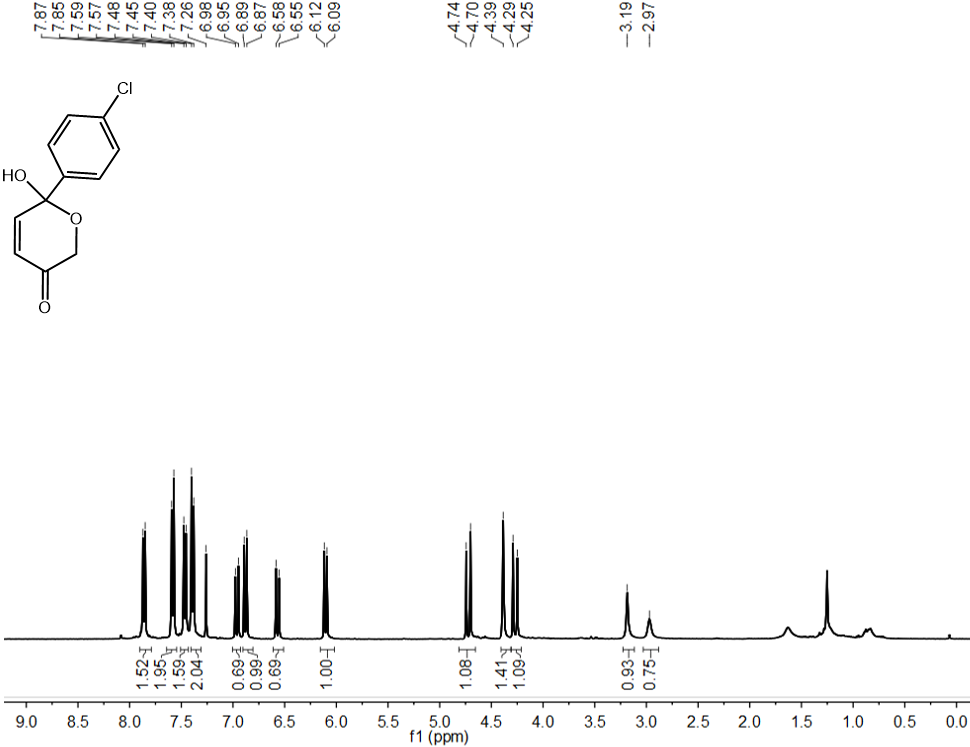

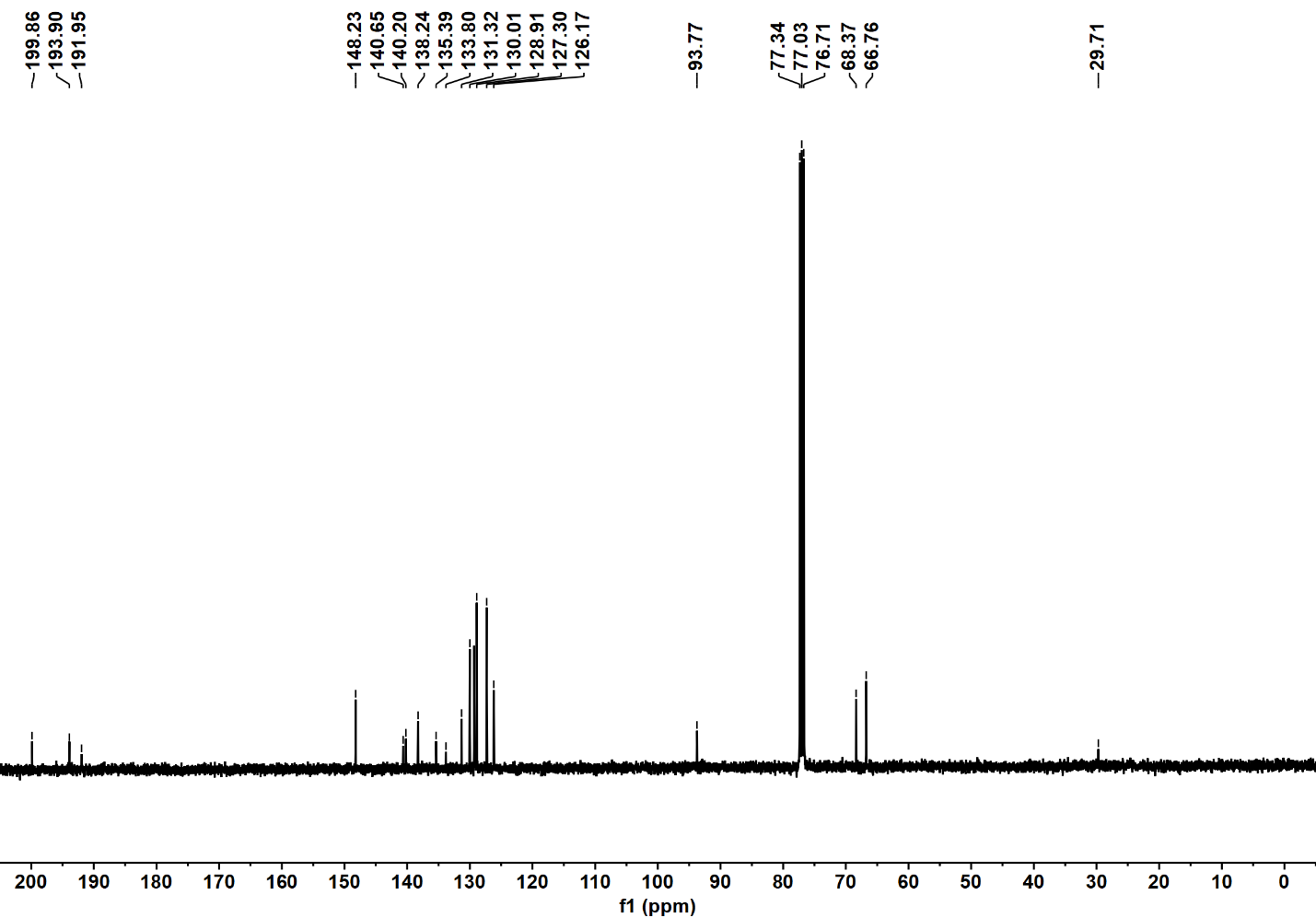


**Ethyl 2-(6-hydroxy-3-oxo-3,6-dihydro-2H-pyran-2-yl)acetate (2l)** Followed Method B from 1l (96 mg, 0.50 mmol) and purified using preparative chromatography to give 79 mg (76% yield) of 2l as a colorless oil.  ^1^H NMR (400 MHz, CDCl_3_) mixture of isomers: δ 7.01 – 6.89 (m, 1H), 6.21 – 6.09 (m, 1H), 5.71 (d, *J* = 2.8 Hz, 0.3H), 5.61 (d, *J* = 4.2 Hz, 0.7H), 5.29 (d, *J* = 1.4 Hz, 0.2H), 5.00 (dd, *J* = 7.7, 4.0 Hz, 0.8H), 4.71 (s, 0.6H), 4.62 – 4.52 (m, 0.3H), 3.69 (d, *J* = 3.9 Hz, 3H), 3.00 (ddd, *J* = 16.8, 8.6, 4.2 Hz, 1H), 2.84 – 2.69 (m, 1H).


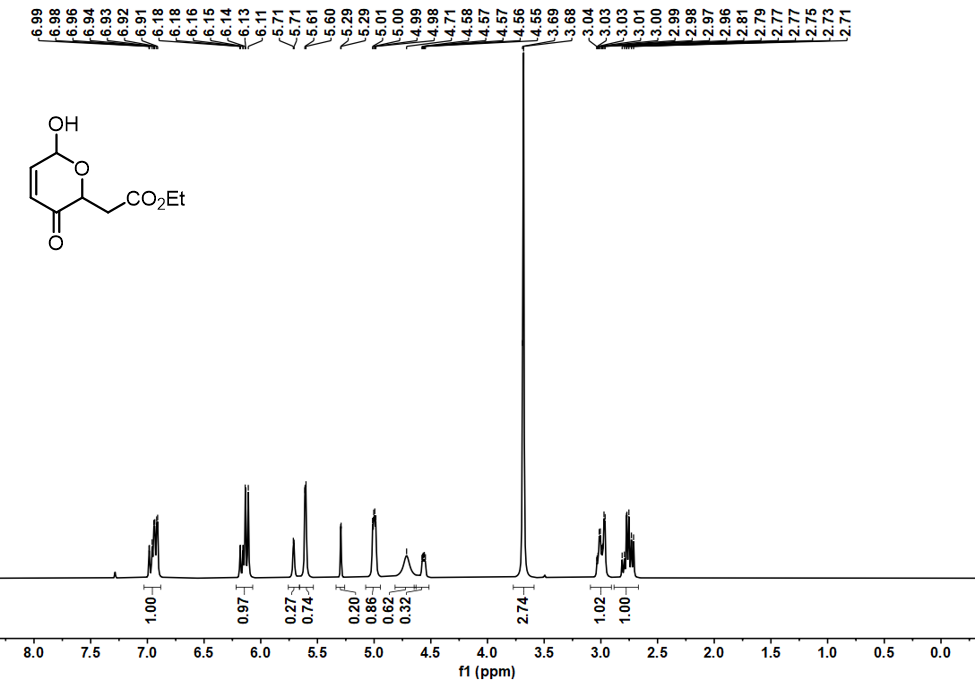


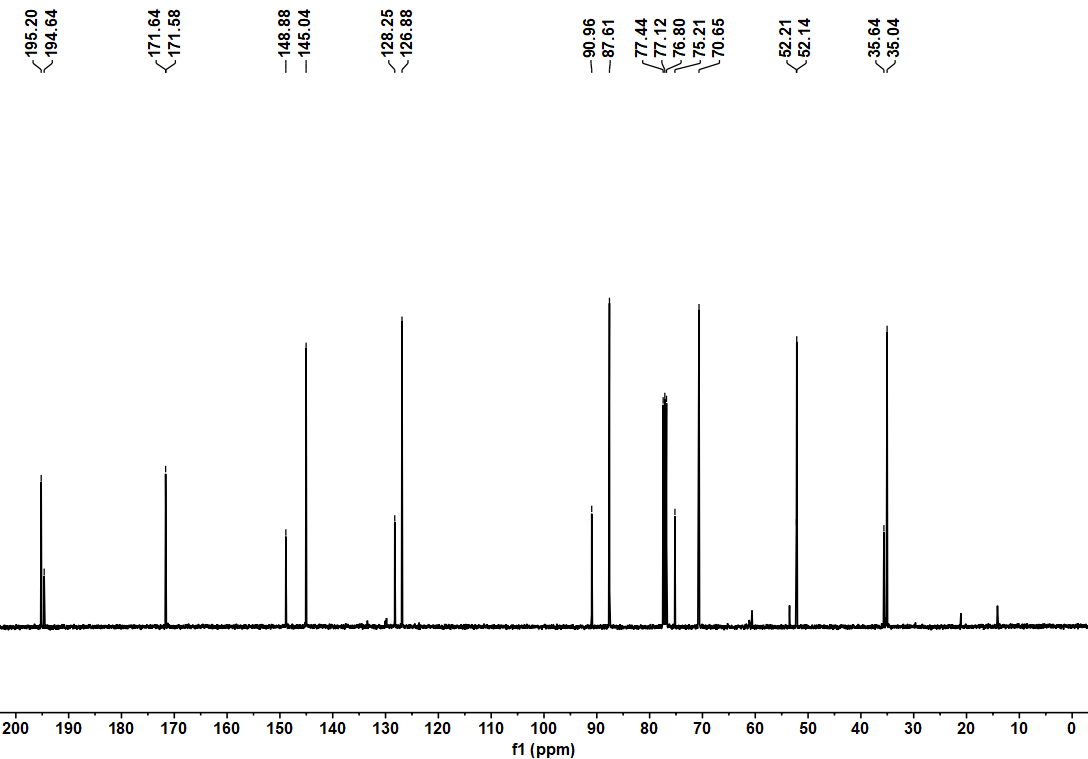


**2-(But-3-en-1-yl)-6-hydroxy-2H-pyran-3(6H)-one (2m)** Followed Method B from 1m (96 mg, 0.50 mmol) and purified using preparative chromatography to give 79 mg (76% yield) of 2m as a colorless oil.  ^1^H NMR (400 MHz, CDCl_3_) mixture of isomers: δ 6.99 – 6.88 (m, 1H), 6.15 (dd, *J* = 17.1, 10.3 Hz, 1H), 5.82 (ddt, *J* = 16.9, 9.9, 6.6 Hz, 1H), 5.68 (d, *J* = 4.6 Hz, 1H), 5.08 – 4.94 (m, 2H), 4.59 (dd, *J* = 8.1, 3.8 Hz, 0.7H), 4.19 – 4.07 (m, 0.3H), 3.51 (d, *J* = 7.1 Hz, 0.3H), 3.26 (d, *J* = 5.3 Hz, 0.7H), 2.20 – 2.04 (m, 2H), 2.04 – 1.87 (m, 1H), 1.86 – 1.70 (m, 1H), 1.56 (dtt, *J* = 17.6, 13.2, 6.7 Hz, 2H).


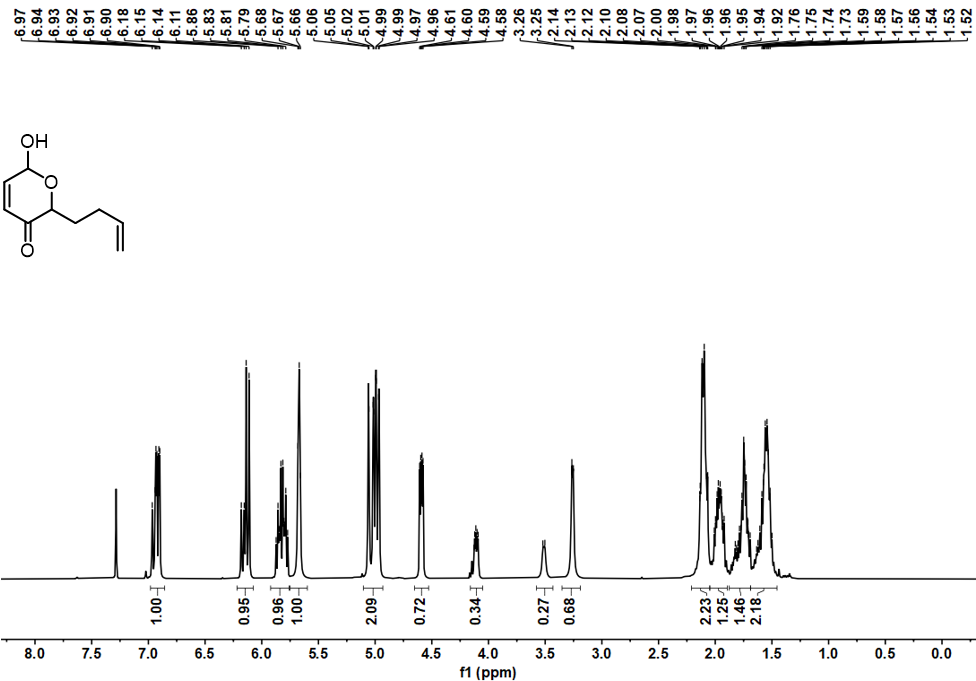


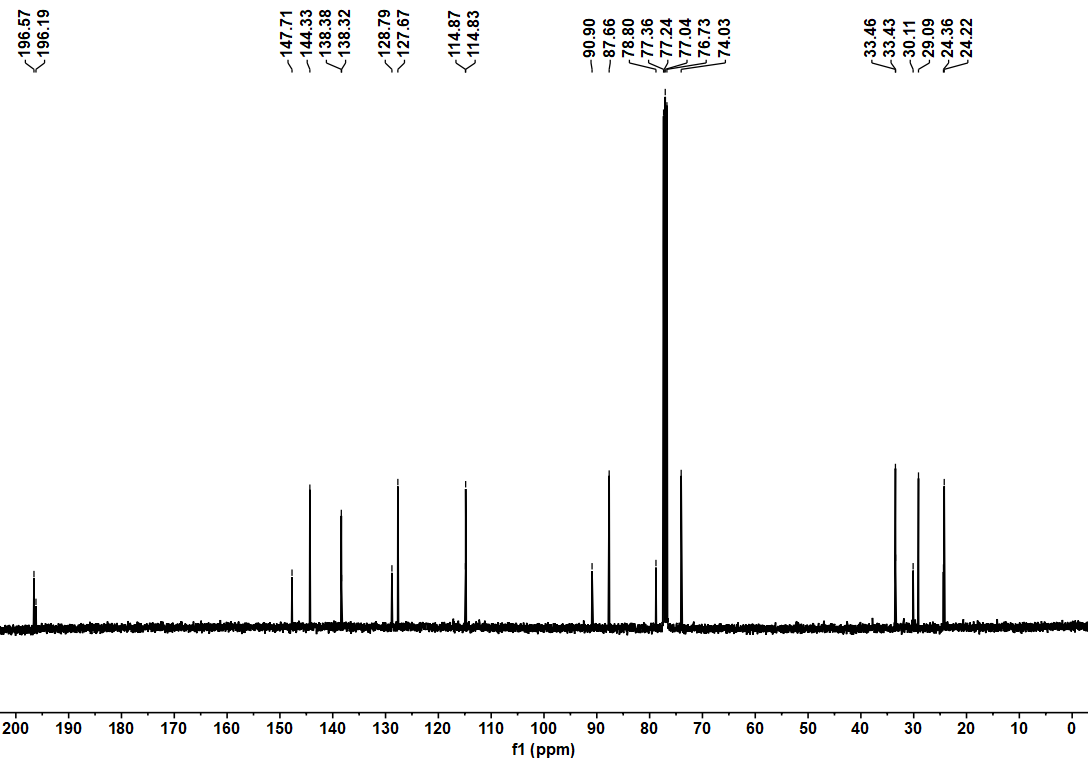


**Cartesian coordinates of intermediates in Figure S6**

**Intermediate 1**

Multiplicity = 2

**
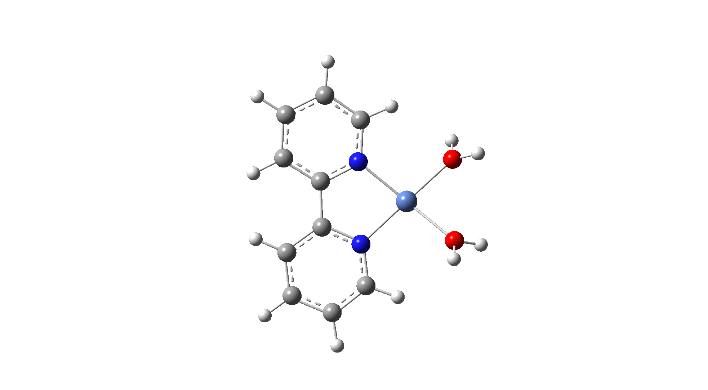
**

C -2.66548 0.03629 0.06277

C -3.49780 -1.07515 0.13540

C -2.91429 -2.34228 0.15099

C -1.52400 -2.46031 0.09809

C -0.74035 -1.30906 0.02600

N -1.32471 -0.07943 0.00716

H -3.53225 -3.23278 0.20767

H -3.06684 1.04365 0.05423

H -4.57311 -0.94427 0.18083

H -1.06565 -3.44131 0.12014

C 0.74044 -1.30902 -0.02598

C 1.52416 -2.46021 -0.09805

C 2.91444 -2.34210 -0.15097

H 1.06587 -3.44125 -0.12005

C 2.66549 0.03645 -0.06283

C 3.49787 -1.07494 -0.13543

H 3.53246 -3.23257 -0.20763

H 3.06678 1.04384 -0.05434

H 4.57317 -0.94399 -0.18090

N 1.32472 -0.07935 -0.00718

Ni -0.00004 1.42484 -0.00001

O 1.25893 2.92738 0.64850

O -1.25912 2.92735 -0.64843

H 1.80032 2.90550 1.45246

H 1.45065 3.75899 0.18894

H -1.80033 2.90557 -1.45252

H -1.45116 3.75877 -0.18867

**Intermediate 2**

Multiplicity = 1


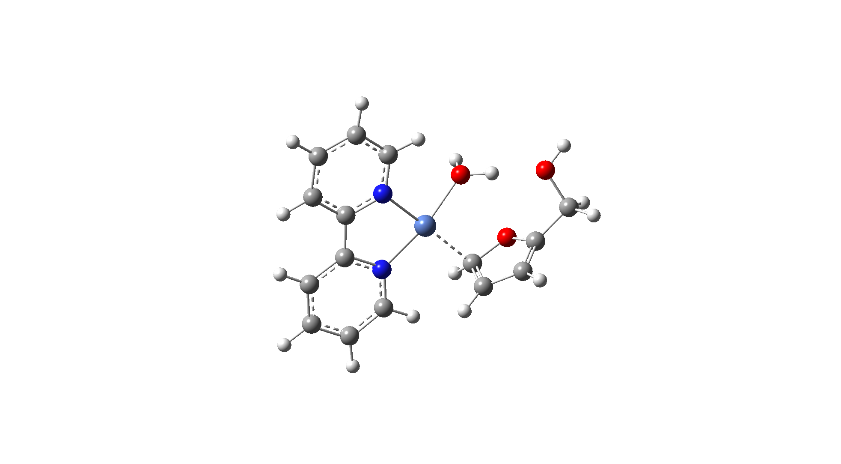


C -0.97265 2.69097 -0.00764

C -2.03237 3.56656 0.21786

C -3.31299 3.04508 0.38662

C -3.49721 1.66278 0.33078

C -2.39786 0.83786 0.10207

N -1.15459 1.36018 -0.06922

H -4.15938 3.70068 0.56405

H 0.04023 3.04511 -0.14221

H -1.84412 4.63345 0.25936

H -4.48483 1.24057 0.47101

C -2.44086 -0.62741 0.03279

C -3.61033 -1.38403 0.10413

C -3.54297 -2.77238 0.01262

H -4.56874 -0.89364 0.22247

C -1.16669 -2.56987 -0.21428

C -2.29728 -3.37561 -0.15141

H -4.44739 -3.36984 0.06433

H -0.19476 -3.02612 -0.34106

H -2.19112 -4.45146 -0.23320

N -1.22252 -1.22525 -0.12162

Ni 0.24252 0.02649 -0.29764

O 1.50766 1.44613 -0.74141

H 1.57465 1.55464 -1.70369

H 2.44486 1.53130 -0.40059

O 2.96993 -0.85934 -0.78692

C 1.77115 -1.47456 -0.44832

C 1.80264 -1.70837 0.94579

C 2.97381 -1.11845 1.44645

C 3.64072 -0.60867 0.34679

H 1.37671 -2.09043 -1.24875

H 1.05427 -2.23291 1.52446

H 3.29053 -1.05178 2.47773

C 4.76039 0.36991 0.21948

H 5.35244 0.39366 1.13879

H 5.40526 0.11776 -0.62963

O 4.07561 1.62109 0.00272

H 4.71006 2.34694 -0.09053

**Intermediate 3**

Multiplicity = 2


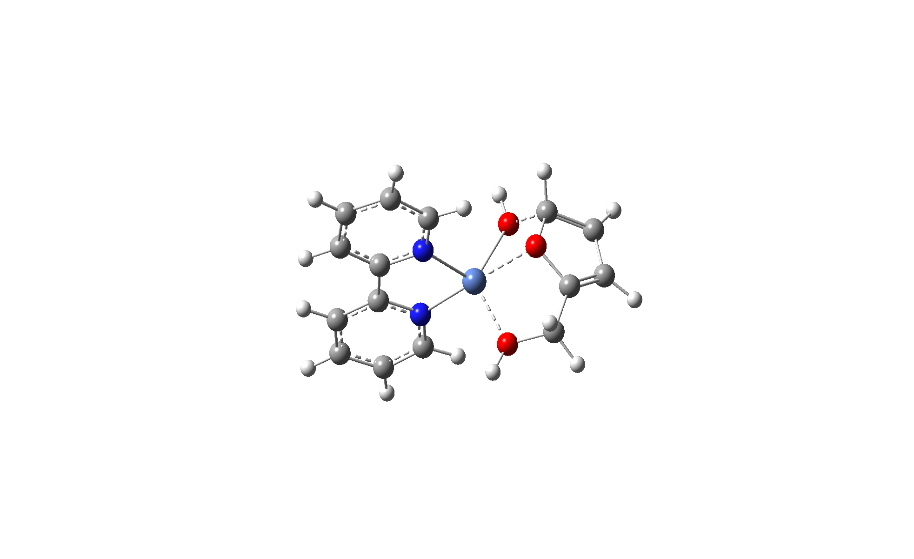


C -0.58703 2.63208 0.05097

C -1.59384 3.59229 0.03682

C -2.91975 3.16203 -0.01389

C -3.19917 1.79499 -0.04720

C -2.14599 0.87900 -0.03073

N -0.86004 1.31652 0.01674

H -3.73223 3.88149 -0.02627

H 0.46171 2.90882 0.09168

H -1.33944 4.64577 0.06523

H -4.22727 1.45651 -0.08405

C -2.32015 -0.59510 -0.05845

C -3.55813 -1.23622 -0.11908

C -3.60734 -2.63109 -0.13847

H -4.47760 -0.66502 -0.15222

C -1.21657 -2.66484 -0.03764

C -2.41987 -3.36115 -0.09711

H -4.56523 -3.13914 -0.18557

H -0.26500 -3.18642 -0.00355

H -2.41870 -4.44518 -0.11050

N -1.17087 -1.32082 -0.01926

Ni 0.48173 -0.18131 0.03467

O 1.58925 -0.35734 -1.86530

H 1.39184 -0.29041 -2.81328

O 2.35605 0.76557 -0.10377

C 2.73642 0.43531 -1.46775

C 3.99796 -0.35110 -1.31233

C 4.19074 -0.60887 0.03139

C 3.20005 0.03891 0.76361

H 2.78792 1.35121 -2.06195

H 4.60734 -0.67782 -2.14423

H 4.97878 -1.21251 0.46390

C 2.64217 -0.05559 2.12990

H 3.21513 -0.75555 2.74191

H 2.58600 0.91888 2.62824

O 1.27204 -0.56643 1.93437

H 0.78887 -0.60217 2.77492

**Intermediate 4**

Multiplicity = 2


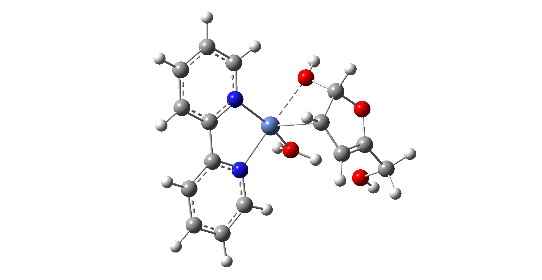


C 1.91113 -2.45020 -0.28213

C 3.19490 -2.94577 -0.07780

C 4.21366 -2.04342 0.22457

C 3.91829 -0.68250 0.30716

C 2.60982 -0.24808 0.08721

N 1.62342 -1.13842 -0.20008

H 5.22844 -2.39033 0.39088

H 1.08260 -3.10803 -0.51792

H 3.38445 -4.01024 -0.15700

H 4.70618 0.02507 0.53317

C 2.19919 1.17875 0.12362

C 3.06609 2.23304 0.41884

C 2.58245 3.54165 0.40034

H 4.10573 2.04788 0.65839

C 0.43232 2.67834 -0.19671

C 1.24394 3.77302 0.08420

H 3.24735 4.36901 0.62659

H -0.61305 2.79468 -0.46085

H 0.83484 4.77645 0.05134

N 0.89671 1.41769 -0.17112

Ni -0.17344 -0.26221 -0.56954

O -1.24770 -2.16811 -0.64504

H -1.75725 -2.69862 -1.28093

O -3.24134 -1.42727 0.31791

C -1.93608 -2.00045 0.57825

C -1.20844 -0.92675 1.36122

C -2.14442 0.08138 1.61651

C -3.31652 -0.24003 0.93522

H -2.09588 -2.95598 1.08945

H -0.31935 -1.13213 1.94598

H -1.99382 0.97751 2.20370

C -4.51135 0.60167 0.61948

H -5.39805 -0.03269 0.51817

H -4.67713 1.33890 1.41198

O -4.17332 1.24190 -0.62385

H -4.97139 1.60067 -1.03876

O -1.69188 0.69733 -1.63338

H -2.62043 0.90252 -1.36460

H -1.62383 0.82768 -2.58897

**Intermediate 5**

Multiplicity = 1


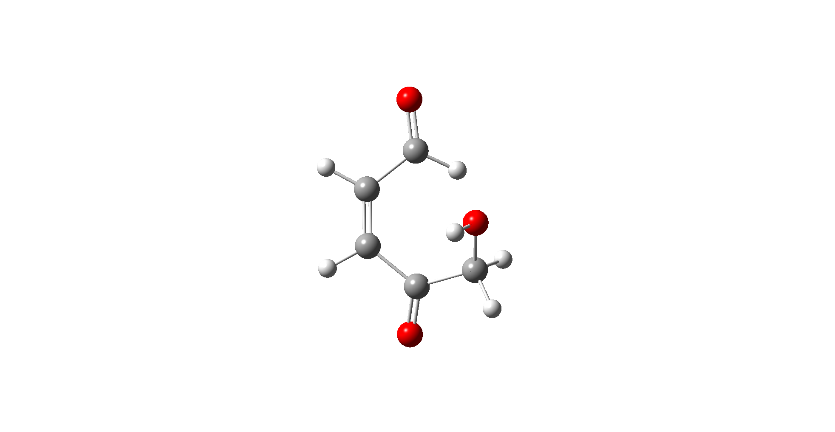


C -1.930856116465 -0.054059750227 -0.783494445635

C -1.004841499138 -0.959796431984 -0.060416818469

C 0.292654703220 -0.753748109753 0.233026287219

C 1.166307387061 0.427542211491 -0.044073413304

C 0.631526226184 1.865818160224 -0.013500597391

O -0.591779972181 2.059456622361 0.668533377239

O -3.113544256330 -0.317968005131 -0.888984805941

O 2.353080141375 0.243893312744 -0.260617770092

H -1.503038706276 0.849298398517 -1.249646213838

H -1.459351793855 -1.912290115556 0.205535730358

H 0.855697765499 -1.568666935030 0.683761236490

H 0.464400072420 2.182559227823 -1.050380369383

H 1.446200321371 2.488590975084 0.379789321893

H -0.449454272884 1.892370439438 1.609468480853

**Cartesian coordinates of intermediates in Figure S7**

**Intermediate 2**

Multiplicity = 2


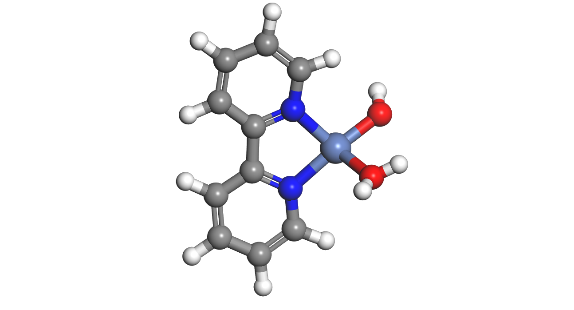


C 2.59578 0.25677 0.03151

C 3.25099 1.47748 0.15253

C 2.49368 2.64932 0.18752

C 1.09990 2.57630 0.11742

C 0.47946 1.33455 0.00207

N 1.24900 0.20434 -0.05343

H 2.98082 3.61542 0.27804

H 3.13204 -0.68354 0.00454

H 4.33397 1.49502 0.21310

H 0.50278 3.47964 0.16704

C -0.97394 1.09988 -0.03488

C -1.96091 2.08111 -0.02241

C -3.30541 1.69492 -0.05144

H -1.69742 3.13215 0.00506

C -2.60913 -0.59991 -0.10384

C -3.63748 0.34038 -0.09220

H -4.08488 2.45052 -0.04234

H -2.81070 -1.66683 -0.13562

H -4.66943 0.00777 -0.11718

N -1.31969 -0.21863 -0.07941

Ni 0.20982 -1.40508 -0.25103

O -0.15275 -2.59440 1.31767

O 1.44683 -2.61723 -0.76372

H -0.44200 -2.32960 2.20508

H 0.44179 -3.36064 1.39849

H 1.98248 -2.46924 -1.56676

**Intermediate 3**

Multiplicity = 2

**
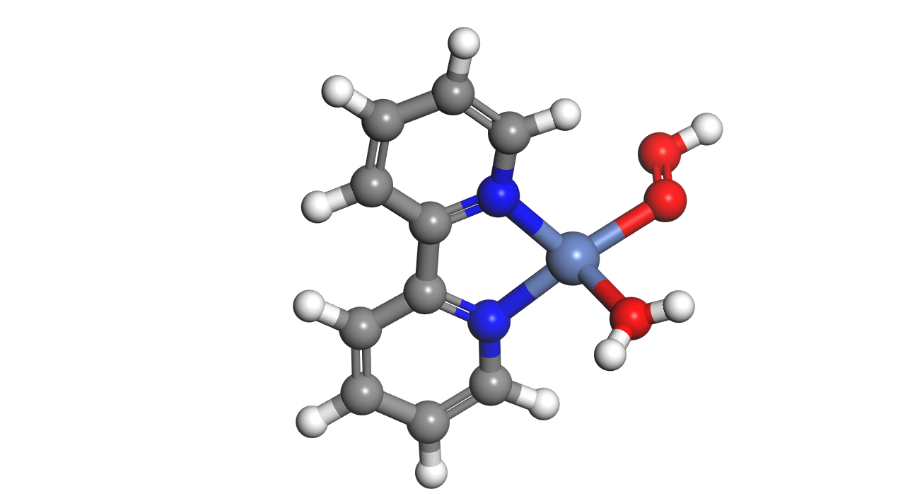
**

C 1.69828 1.90705 0.18637

C 1.47297 3.27946 0.17471

C 0.15953 3.73634 0.06359

C -0.88589 2.81499 -0.03109

C -0.60127 1.45010 -0.01413

N 0.68769 1.02277 0.09198

H -0.05386 4.80053 0.05217

H 2.70045 1.50030 0.27346

H 2.30911 3.96540 0.25160

H -1.90734 3.16561 -0.11417

C -1.62328 0.37959 -0.10398

C -2.99065 0.60438 -0.25502

C -3.86023 -0.48627 -0.33538

H -3.38350 1.61197 -0.31448

C -1.97704 -1.94680 -0.11027

C -3.34974 -1.78270 -0.26295

H -4.92632 -0.32077 -0.45481

H -1.53039 -2.93468 -0.04884

H -3.99476 -2.65203 -0.32439

N -1.14096 -0.89254 -0.03386

Ni 0.83833 -0.96244 0.09441

O 1.16665 -1.79247 1.90175

O 2.74791 -1.53174 -0.39879

H 0.55165 -1.71238 2.64672

H 2.02923 -2.06283 2.25406

O 3.04354 -1.35311 -1.67452

H 3.98450 -1.64290 -1.77026

**Intermediate 4**

Multiplicity = 1


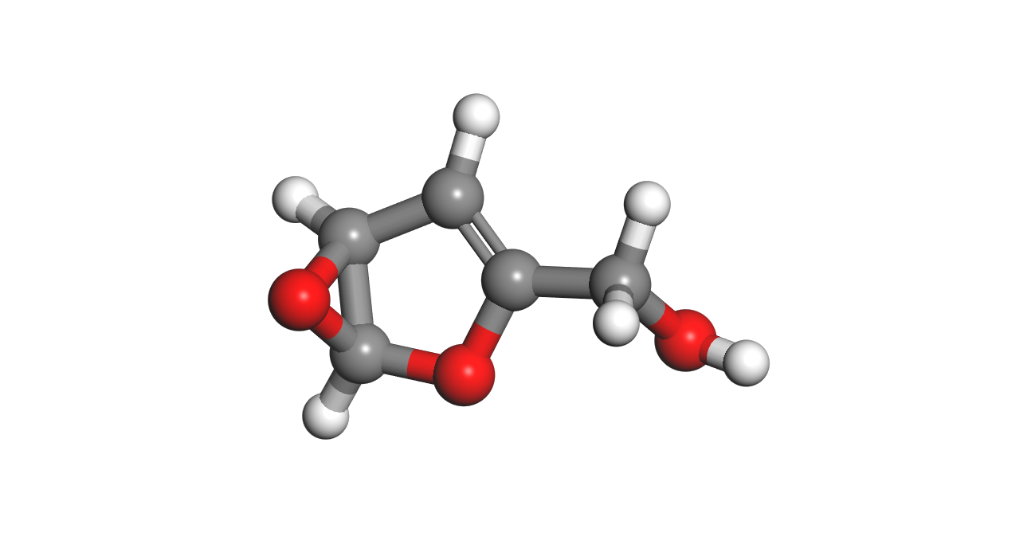


O -0.05862 -0.99880 -0.00923

C -1.42956 -0.83257 -0.28529

C -1.70988 0.60668 -0.44608

C -0.40074 1.24950 -0.20567

C 0.47701 0.26995 0.08224

H -0.18280 2.30693 -0.24083

C 1.90312 0.33969 0.51085

H 2.17976 1.39980 0.61057

H 1.98772 -0.12279 1.50606

O 2.71357 -0.33635 -0.44351

H 3.55341 -0.54859 -0.01787

O -2.18216 -0.18619 0.70853

H -2.49754 1.03101 -1.05991

H -1.86261 -1.69518 -0.78055

**Supplementary References**

[1] Liu, Y., Zhang, S., Miao, Q., Zheng, L., Zong, L. & Cheng, Y. Fluorescent chemosensory conjugated polymers based on optically active polybinaphthyls and 2,2‘-bipyridyl units. *Macromolecules* **40**, 4839-4847 (2007).

[2] Li, M., Dong, J., Huang, X., Li, K., Wu, Q., Song, F. & You, J. Nickel-catalyzed chelation-assisted direct arylation of unactivated C(sp^3^)–H bonds with aryl halides. *Chem. Commun.* **50**, 3944-3946 (2014).

[3] Wang, H.-Y., Yang, K., Bennett, S. R., Guo, S.-r. & Tang, W. Iridium-catalyzed dynamic kinetic isomerization: expedient synthesis of carbohydrates from Achmatowicz rearrangement products. *Angew. Chem. Int. Ed.* **54**, 8756-8759 (2015).

[4] Wencel-Delord, J. & Colobert, F. A remarkable solvent effect of fluorinated alcohols on transition metal catalysed C–H functionalizations. *Org. Chem. Front.* **3**, 394-400 (2016).

[5] Ronn, M., Lim, N.-K., Hogan, P., Zhang, W.-Y., Zhu, Z. & Dunwoody, N. An expedient route to 3-methoxy-2-furaldehyde. *Synlett* **2012**, 134-136 (2012).

[6] Christoforow, A., Wilke, J., Binici, A., Pahl, A., Ostermann, C., Sievers, S. & Waldmann, H. Design, synthesis, and phenotypic profiling of pyrano-furo-pyridone pseudo natural products. *Angew. Chem. Int. Ed.* **58**, 14715-14723 (2019).

[7] Fürstner, A. & Nagano, T. Total Syntheses of ipomoeassin B and E. *J. Am. Chem. Soc.* **129**, 1906-1907 (2007).
